# Post-Print Annealing of FDM-Printed Polylactic Acid: Mapping Strength, Crystallinity, and α′/α Polymorph Composition via a Replicated Taguchi L9 Design

**DOI:** 10.3390/polym18111338

**Published:** 2026-05-28

**Authors:** Walid M. Shewakh, Majed H. Moosa, Zainab Hussein Mohsein, Osama M. Irfan

**Affiliations:** 1 Department of Industrial Engineering, College of Engineering and Computer Sciences, Jazan University, Jazan 82817, Saudi Arabia; mmoosa@jazanu.edu.sa; 2 College of Production Engineering and Metallurgy, University of Technology, Baghdad 10066, Iraq; 3 Department of Mechanical Engineering, College of Engineering, Qassim University, Buraydah 51452, Saudi Arabia

**Keywords:** polylactic acid, fused deposition modeling, annealing, tensile strength, Taguchi method, DSC, XRD, α′/α polymorphism, crystallinity, multivariate regression, ANOVA

## Abstract

Fused deposition modeling (FDM) of polylactic acid (PLA) produces parts whose weak interlayer bonding and low as-printed crystallinity limit their tensile performance. This work used a Taguchi L9 orthogonal array with five replicates per cell (n = 5; N = 45 annealed specimens plus five non-annealed controls) to study how annealing temperature (70, 80, and 90 °C) and holding time (40, 60, and 80 min) change the tensile response of a commercial PLA grade (eSUN PLA+) printed on a desktop FDM machine. Differential scanning calorimetry (DSC) and X-ray diffraction (XRD) were used in parallel to measure total crystallinity, and XRD was deconvoluted to estimate the α′/α polymorph fractions; the DSC α′→α exothermic shoulder was used as an independent cross-check. Every annealed condition exceeded the non-annealed baseline ultimate tensile stress (UTS) of 39.75 ± 1.28 MPa. The optimum, 47.00 ± 0.97 MPa at 70 °C/60 min, gave an 18.2% gain. Total crystallinity rose from 8.6% (DSC baseline) to 41.8% (DSC, 90 °C/80 min), with DSC and XRD ranking the conditions consistently. ANOVA confirmed both temperature (30.0% contribution) and time (24.2%) as significant at α = 0.05. The new contribution is a combined strength–crystallinity–polymorph map for desktop FDM-printed PLA: the best-performing specimens are dominated by the disordered α′ form, while the stiffer but weaker high-temperature specimens shift toward α. A partial least squares regression on all 50 specimens supports the polymorph-composition role beyond what total crystallinity alone explains. The practical conclusion is that moderate annealing just above the glass transition gives the best balance of crystal content, polymorph character, and geometric stability for FDM-printed PLA.

## 1. Introduction

Additive manufacturing (AM) builds parts layer by layer from a digital model [1]. Fused deposition modeling (FDM) is by far the most common AM method in small labs and workshops because it is inexpensive, easy to operate, and compatible with a wide range of thermoplastic filaments [2]. A heated nozzle melts the feed filament and deposits it along a toolpath to draw each layer. The trade-off is well documented: mechanical properties of FDM parts usually fall short of injection-molded parts made from the same polymer [3]. Weak interlayer bonding, residual porosity, and anisotropy from the layer-wise build are the main causes. Other AM techniques, such as stereolithography, selective laser sintering, and material jetting, each have their own strengths and limits [4,5]. What sets FDM apart is its flexibility with thermoplastic filaments, from commodity grades such as PLA and ABS to engineering materials such as PEEK and polycarbonate [6].

Polylactic acid (PLA) is one of the most widely used filaments for FDM. It is a semi-crystalline thermoplastic produced from renewable resources, and its biodegradability makes it attractive for sustainability-minded applications [7]. PLA is the general name for the polymer; PLLA refers specifically to poly(L-lactic acid), the L-stereoisomer. Commercial PLA filaments are usually predominantly PLLA with a small fraction of D-isomer that controls crystallization rate and upper use temperature; the polymorph literature on PLA crystallization is almost entirely on PLLA, which is why we use the PLLA label whenever we cite that body of work. PLA has a fairly low melting point (about 170–180 °C), good stiffness, and a clean surface finish. Its weaknesses are brittleness, modest heat resistance, and a low as-printed crystallinity that limits mechanical performance. The glass transition temperature (*T*_g_) of PLA lies near 55–65 °C, and its cold crystallization temperature (*T*_cc_) falls between 80 and 120 °C depending on grade and thermal history [8]. The window between *T*_g_ and *T*_cc_ is where polymer chains can rearrange into crystalline regions without fully melting.

An important detail, often skipped in the FDM literature, is that PLA crystallizing below about 110 °C does so predominantly in the disordered α′ modification rather than the thermodynamically stable α form [9,10]. The α′ phase has looser chain packing, broader XRD reflections, and weaker intermolecular interactions than α, and a solid–solid α′→α transition appears on subsequent heating near 150 °C [11,12]. The two polymorphs also have measurably different enthalpies of melting: Righetti and co-workers reported Δh°_m_ values of about 107 J/g for α′ at 150 °C versus 143 J/g for α at 180 °C [13]. Because the polymorph that forms influences stiffness, strength, and barrier properties [14], any mechanistic account of annealed FDM-PLA should consider not only total crystallinity but also which crystal form is produced.

Thermal annealing is a simple heat treatment: a part is held at an elevated temperature for a set period, then cooled. Given enough mobility and time, PLA chains organize into ordered lamellae, which generally raises stiffness, tensile strength, and thermal stability [15]. Several groups have studied annealing of FDM-printed PLA. Wach et al. [15] showed that holding PLA above Tg raised flexural strength by 11–17%. Akhoundi et al. [16] reported that combined nozzle-temperature tuning and post-print annealing pushed tensile strength and modulus of a high-temperature PLA to 67.4 MPa and 5.65 GPa, respectively. Jayanth et al. [17] tested heat treatment of FDM-printed PLA and observed significant increases in tensile strength, modulus, and hardness, with printing orientation strongly influencing the magnitude of the gain. Kartal and Kaptan [18] reported a 48% rise in tensile stress after annealing at 85 °C for 90 min, together with a 78% rise in elastic modulus. Kotsilkova et al. [19] found that graphene-filled PLA annealed at 80 °C gained about 11% in tensile strength, with a clear decoupling between stiffness and strength. Pastorek and Kovalcik [20] confirmed that the amorphous-to-crystalline transition near 100 °C improved both thermal and mechanical properties in PLA. Basgul et al. [21] reported that FFF-printed PEEK lumbar cages annealed at 200 °C and 300 °C did not show clear mechanical gains, a useful reminder that the annealing response depends on material and geometry. Stojković et al. [22] varied layer height and annealing parameters on desktop PLA and found the best combination near 90 °C and 60 min. Simmons et al. [23] linked nucleation, annealing, and crystallinity in PLA, showing that controlled nucleation is what turns heat input into useful mechanical gains. Bhandari et al. [24] demonstrated similar effects in carbon-fiber-reinforced PETG/PLA composites.

Beyond strength numbers, a second strand of the PLA literature looks specifically at polymorph evolution. Zhang et al. [11] showed that PLLA crystallized below about 100 °C forms the α′ phase, while above about 110 °C, the more compact α form grows. Kawai et al. [12] mapped how the α′ fraction falls as annealing temperature rises. Di Lorenzo and co-workers [9,10,14] extended this picture and showed that the α′/α ratio directly affects mechanical and barrier properties in bulk PLLA. Androsch, Schick, and Di Lorenzo [25] characterized the melting and reorganization kinetics of conformationally disordered α′ crystals, showing that reorganization into α can be suppressed at fast heating rates. None of that work used FDM-printed material, so carrying the polymorph lens over to desktop FDM PLA is still a useful step.

Despite a growing body of FDM annealing work, several gaps remain. Many studies tested a narrow parameter window or a single temperature. Few cross-checked mechanical gains against crystallinity measured by two independent methods (DSC and XRD), and, to the authors’ knowledge, none have tracked α′/α polymorph composition alongside the mechanical response on desktop FDM PLA. Another common gap is limited replication: single specimens per condition do not support reliable statistical inference.

A further methodological point deserves more emphasis than it has received in prior FDM-PLA annealing studies. FDM deposition is itself a strongly non-isothermal process. Each newly deposited bead cools at a position-dependent rate that depends on the temperature of the layer beneath it, the build plate temperature, the cooling-fan flow field, and the local toolpath density. As a consequence, the as-printed state already carries a non-uniform thermal history before any annealing step is applied; bead boundaries and bulk material can sit in different polymorph and crystallinity states even within a single part. This is a plausible additional contributor to the scatter in reported annealing gains across studies, alongside the narrow parameter windows and single-replicate designs already noted. The replicated design used here cannot remove this source of variability, but can at least bound it: the within-condition replicate scatter we report (standard deviations of 0.66 to 1.20 MPa, coefficients of variation of 1.5 to 2.8%) captures the combined effect of print-stage non-isothermal variability and anneal-stage variability, and it is small enough to support the factor-effect inferences that follow.

The present study addresses these gaps. A Taguchi L9 orthogonal array with n = 5 replicates per condition was used to quantify the effects of annealing temperature (70, 80, and 90 °C) and time (40, 60, and 80 min) on the ultimate tensile stress (UTS) of FDM-printed PLA. Crystallinity was measured by both DSC and XRD, with the XRD patterns further analyzed to estimate the α′/α phase ratio at each condition. ANOVA with the interaction term retained, signal-to-noise (S/N) ratio analysis, and stress–strain characterization were combined to identify the optimal combination of parameters and to explain the mechanical response through crystallinity and polymorph data. A multivariate analysis (partial least squares regression and a random forest model) on the full 50-specimen dataset was added to quantify how much variance in UTS is uniquely explained by temperature, time, total crystallinity, and α′ fraction simultaneously. The central contribution of this work is the combined strength–crystallinity–polymorph map for desktop FDM-printed PLA, which has not, to the authors’ knowledge, been reported previously.

## 2. Materials and Methods

### 2.1. Material

Commercial PLA filament (eSUN PLA+, Shenzhen Esun Industrial Co., Shenzhen, China) with a 1.75 mm diameter was used for all specimens. The “+” designation indicates a modified formulation, which for typical eSUN PLA+ lots contains an impact modifier and may also contain a nucleating agent, both at concentrations generally below a few percent. The manufacturer does not disclose the exact additive package for individual lots. Three implications follow. First, the reference enthalpy of fusion of 100% crystalline PLLA homopolymer (Δh°m = 93.6 J/g, commonly cited [8]) is used here as a convention to compute DSC crystallinity, but the absolute crystallinity values should be read as internally consistent measurements rather than as absolute PLLA numbers. Recent work [13] has argued for polymorph-specific Δh°m values of 107 J/g for α′ and 143 J/g for α; recomputing with those values would shift the crystallinity numbers but not the relative ranking across conditions. Second, nucleating agents can bias the α′/α distribution, so absolute polymorph fractions reported here may differ from those measured on PLLA homopolymer at the same temperature. Third, the cross-comparisons between conditions in this study remain valid because the same filament lot, printer settings, and test protocol were used throughout. Key manufacturer-reported properties are listed in Table 1. Molecular weight and the exact L/D stereoisomer ratio were not measured in-house.

### 2.2. Specimen Design and Fabrication

**Figure 1:**
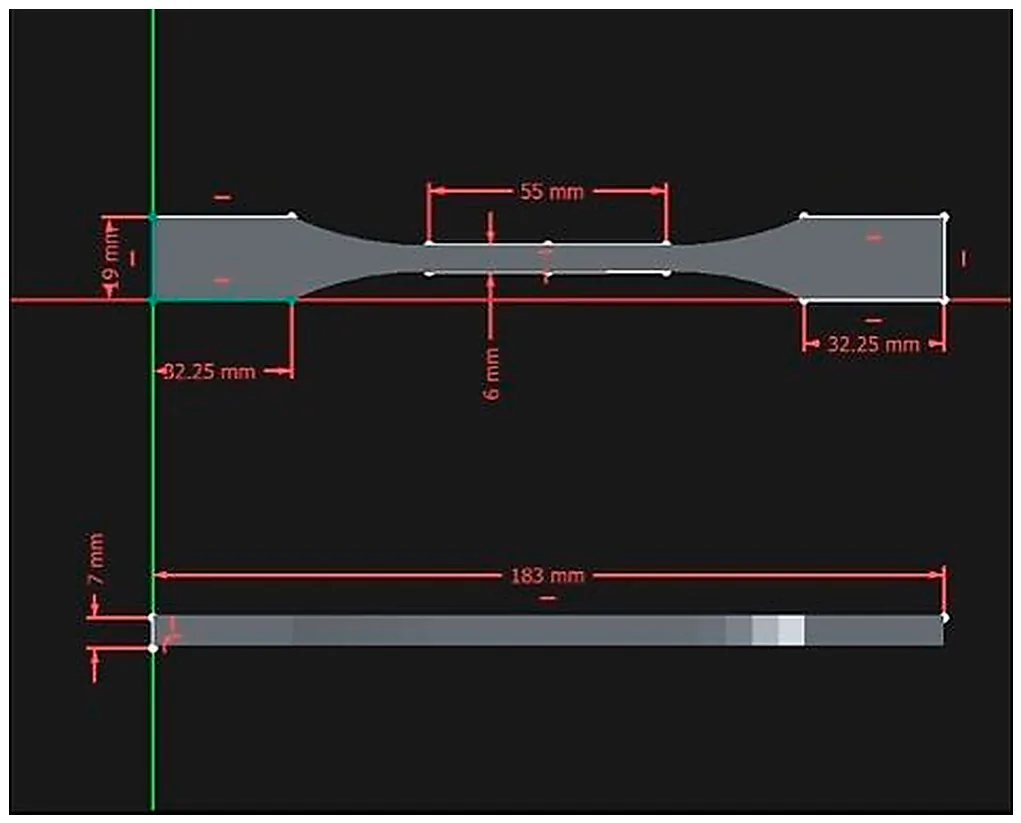
Dog-bone tensile specimen geometry (ASTM D638 Type II). Gauge length of 55 mm, width at gauge of 6 mm, grip width of 19 mm, total length of 183 mm, and thickness of 7 mm.

Dog-bone tensile coupons were designed in SolidWorks 2022 per ASTM D638-22 Type II [26], with a nominal gauge length of 55 mm, thickness of 7 mm, and width of 6 mm (Figure 1). STL files were sliced and printed on a Creality Ender-3 (Shenzhen Creality 3D Technology Co., Ltd., Shenzhen, China) FDM machine. Printing parameters are listed in Table 2, and measured specimen dimensions (mean ± SD across five replicates) are given in Table 3. Dimensions were verified with a Vernier caliper (resolution 0.02 mm, expanded uncertainty ±0.03 mm at *k* = 2). For the nominal 6 mm × 7 mm cross-section, this translates into a propagated uncertainty in engineering stress of about 0.8%, which is smaller than the observed replicate scatter.

The nozzle temperature was 210 °C for all specimens. A print speed of 120 mm/s was used, which sits at the aggressive end of the usable range for a stock Ender-3 with a 0.4 mm nozzle.

A short preliminary qualification was carried out before the main campaign: five single-wall test cubes were printed at 120 mm/s and sectioned, and optical inspection of the sectioned cubes did not reveal under-extrusion, voids, or stringing at the resolution of the stereomicroscope used, with wall thickness matching the slicer target to within 0.05 mm. Ten dog-bone specimens were then printed as a pilot batch; none were rejected on visual grounds. SEM was not performed at this stage and is a meaningful limitation of the print-quality qualification; SEM fractography of the tested specimens is one of the priorities for the follow-up study (Section 3.10). Chacón et al. [27] reported that moderate-to-high print speeds remain compatible with acceptable tensile properties on desktop FDM machines when layer height and nozzle temperature are held constant, although their highest tested speed was 80 mm/s, so the present 120 mm/s value sits above the range they directly characterized.

This has an important implication for how the absolute numbers in this study should be read. The as-printed baseline UTS obtained here (39.75 MPa) sits at the lower end of literature values for FDM-printed PLA, where 50–60 MPa is common for well-tuned desktop setups at 50–80 mm/s. Part of the offset is likely explained by the elevated print speed and part by the modified PLA+ grade, which is impact-toughened and trades peak tensile stress for ductility. Because the same printing recipe, filament lot, and handling protocol were applied to every specimen, this does not bias the within-study comparisons: all annealing gains are reported as percent changes over the same baseline. Readers should, however, be cautious in comparing the absolute MPa values reported here to those from studies that used 50–80 mm/s and homopolymer PLA.

The cooling fan ran at 100% throughout printing. Rapid cooling suppresses in situ crystallization and leaves the part largely amorphous (baseline DSC crystallinity of 8.6%), so any crystallinity gain measured later can be attributed to annealing rather than to the printing step itself.

Measured thicknesses exceeded the nominal 7.00 mm by 0.2 to 0.7 mm, a common outcome on entry-level FDM machines due to extrusion over-deposition and layer stacking tolerances. All stress values reported later were computed from the actual measured cross-section of each individual specimen, not from nominal dimensions.

Three observations explain the directional pattern in the dimensional offsets and rule out crystallization shrinkage as the dominant contribution. First, the offsets are largest in the build direction (thickness, z) because each printed layer carries a small positive extrusion margin that stacks additively across the 35 layers of the 7 mm cross-section, whereas the in-plane width is set by perimeter passes that are constrained to the slicer toolpath and therefore accumulate less error. The thickness-to-width offset ratio across all conditions sits between 2.4 and 3.7, which is consistent with a stacked-layer rather than an isotropic-volumetric mechanism. Second, the part-to-part scatter within a single condition (thickness SD ≤ 0.11 mm) is much smaller than the consistent positive offset from nominal, which is again the signature of a systematic stacking error rather than random density variation. Third, the same dimensional measurements were repeated after annealing on a matched set of five Exp. 9 (90 °C/80 min) specimens to check for dimensional drift; post-anneal width and thickness changed by less than 0.05 mm on average (below caliper resolution), so in-plane geometric distortion at the worst annealing condition is small and a crystallization-driven density change cannot be the dominant contributor to the offsets, which would otherwise produce a measurable post-anneal shrinkage.

### 2.3. Design of Experiments

A Taguchi L9 orthogonal array was used [28]. The two control factors were annealing temperature (70, 80, and 90 °C) and time (40, 60, and 80 min). The chosen window sits between *Tg* (≈60 °C) and *Tcc* (≈100 °C), where cold crystallization is active but full melting is not. In a single-replicate L9, the error term has only four degrees of freedom, which gives very limited statistical power. The present study used five replicates per L9 condition (n = 5), for a total of 45 annealed specimens plus five non-annealed controls (N = 50). The replicated design raises the available error degrees of freedom to 36 with the interaction term retained, or 40 after pooling a non-significant interaction into error, which gives meaningful F tests for each factor. The experimental matrix is summarized in Table 4.

### 2.4. Annealing Procedure

Annealing was carried out in a calibrated convection oven (Memmert UF30, Memmert GmbH + Co. KG, Schwabach, Germany; ±1 °C temperature uniformity). Specimens were placed flat on a ceramic plate inside the preheated oven. Replicates for each condition were processed in the same oven run to minimize batch-to-batch variability, with specimen positions rotated between runs. After the specified holding time, the oven was switched off, and its door was kept closed; specimens cooled in still oven air to room temperature over about 3–4 h.

Because the cooling rate through the crystallization-sensitive window influences final morphology, a dedicated calibration run was carried out with a K-type thermocouple (Omega SC-TT-K-30, ±0.75% accuracy, Norwalk, CT, USA) embedded in a sacrificial dummy specimen on the oven shelf, logged at 1 Hz from the hold set-point through to ambient. Between the hold temperature and 55 °C, the cooling rate averaged 0.58 ± 0.04 °C/min across three repeat calibration runs performed under otherwise identical oven-cool conditions. This is consistent with the 0.5–0.7 °C/min range cited in common practice for PLA annealing [15,16] and indicates that run-to-run cooling variability is small enough to be absorbed into the reported replicate standard deviations. The main oven runs themselves were not individually logged with the thermocouple, which remains a limitation flagged in Section 3.10.

### 2.5. Tensile Testing

**Figure 2:**
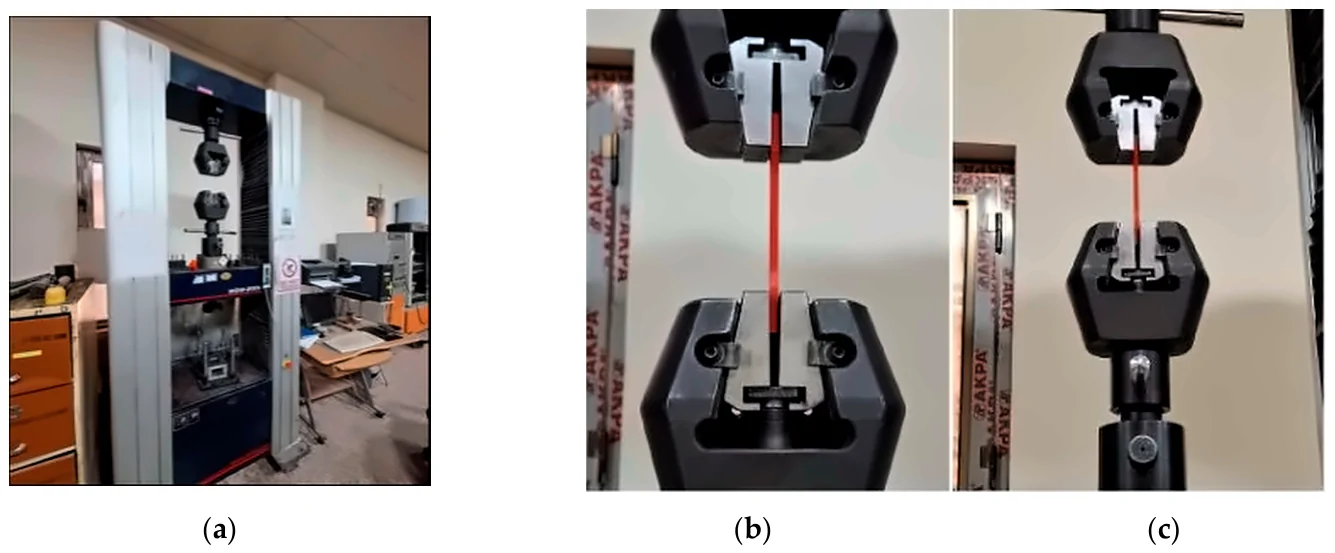
Tensile test setup: (**a**) universal testing machine used for tensile testing; (**b**) specimen in serrated grips before testing; (**c**) specimen after fracture at mid-gauge.

Tensile tests were performed on a universal testing machine per ASTM D638-22 [26] at a crosshead speed of 5 mm/min and a laboratory temperature of 23 ± 2 °C. Specimens were mounted in serrated wedge grips Figure 2. Load and crosshead displacement were recorded digitally at 50 Hz. Ultimate tensile stress, ultimate strain, fracture stress, and fracture strain were extracted from the load–displacement traces after converting to engineering stress and strain using the measured cross-section of each specimen.

Strain was computed from crosshead displacement rather than from a contacting or optical extensometer because no extensometer was available on the machine. Crosshead-based strain overestimates specimen strain because it includes machine compliance and grip take-up. A compliance correction was applied following ASTM E111-17(2025)e1 [29] guidance: the machine compliance was determined from a stiff steel reference specimen of the same grip geometry and subtracted from the raw crosshead trace before computing specimen strain. After this correction, the elastic modulus values reported in Section 3.2 should be read as lower-bound estimates with an uncertainty of roughly ±7%, larger than the ±2% replicate scatter within each condition. The ultimate tensile stress is computed from load and cross-section and is not affected by compliance. Elastic modulus was extracted from the initial linear portion of each compliance-corrected stress–strain curve in the 0.05–0.25% strain range. A sensitivity check using a 0.10–0.40% window gave modulus values within 3% of those reported here, so the extraction window does not materially affect the ranking across conditions.

### 2.6. Differential Scanning Calorimetry (DSC)

DSC analysis was performed on 5–10 mg samples cut from the gauge section of each specimen after tensile testing, taking material from the grip end well away from the fracture zone (one sample per replicate, n = 5 per condition). Samples were sealed in aluminum pans and heated from 30 to 200 °C at 10 °C/min under a 50 mL/min nitrogen purge. Only the first heating scan is reported for crystallinity analysis because that scan carries the combined thermal history of printing plus annealing, which is what the tensile specimens actually experienced. A second-heating scan was run on a subset of three conditions (baseline, Exp. 2, and Exp. 9) to confirm that the intrinsic polymer response is similar across conditions after erasing the thermal history; those scans are not used in the *X*_c_ calculations.

The glass transition temperature (*T*_g_), cold crystallization temperature (*T*_cc_), melting temperature (*T*_m_), and associated enthalpies (Δ*H*_cc_, Δ*H*_m_) were extracted from the first heating scan. The degree of crystallinity (*X*_c_) was computed as [8]:

*X*_c_ = [(Δ*H*_m_ − Δ*H*_cc_)/Δ*h°*_m_] × 100%

where Δ*h°*_m_ = 93.6 J/g is the standard reference enthalpy of fusion for 100% crystalline PLLA [8]. As noted in Section 2.1, the recent literature values based on polymorph-specific measurements give 107 J/g for α′ at 150 °C and 143 J/g for α at 180 °C [13]. Using the 93.6 J/g convention, as is standard in the FDM-PLA literature, allows direct comparison with prior studies; the relative ranking across conditions is unchanged if the polymorph-specific values are used instead. This formulation assumes that any cold-crystallization exotherm observed on heating reflects crystal growth that did not occur during the original thermal treatment. For the most severely annealed specimens, Δ*H*_cc_ approaches zero and the formula collapses to *X*_c_ = Δ*H*_m_/Δ*h°*_m_. A small α′→α exothermic feature near 150–160 °C was observed in some scans and was integrated separately rather than being absorbed into Δ*H*_m_; this feature is discussed in Section 3.5, and its integrated enthalpy is used in Section 3.5 to back out an independent estimate of the α′ fraction that can be cross-checked against the XRD value.

### 2.7. X-Ray Diffraction (XRD)

XRD patterns were collected on a laboratory diffractometer using Cu Kα radiation (λ = 1.5406 Å) at 40 kV and 30 mA, over a 2θ range of 5–40° with a scan rate of 2°/min and step size of 0.02°. One sample was analyzed per replicate (n = 5 per condition). For a subset of samples (one per temperature group), a slower rescan at 0.5°/min across 13–18° 2θ was performed to confirm the polymorph-sensitive peaks were adequately resolved at the main scan rate. The degree of crystallinity from XRD was obtained by fitting each pattern with pseudo-Voigt profiles for the crystalline reflections and a broad Gaussian for the amorphous halo, following the general deconvolution procedure established for semicrystalline polymers by Murthy and Minor [30], taking the ratio of total crystalline peak area to total diffracted area.

The PLA α-form peak positions used as initial fit guesses were 2θ = 14.8°, 16.7°, 19.1°, and 22.4°, cross-checked against both the reference crystal structure of Wasanasuk et al. [31] and recent FDM PLA diffraction patterns [32]. The α′/α polymorph fractions reported later were obtained as follows. Each pattern was fitted with two overlapping pseudo-Voigt components centered near 16.5° (assigned to α′) and 16.7° (assigned to α), with widths constrained to ranges from the literature for each phase [11]. The α′ fraction was then taken as the ratio of the integrated intensity of the 16.5° component to the sum of the two components. The α′ peak is broader and slightly lower in 2θ than the α peak, and in samples dominated by α′, a weak companion reflection appears near 14.8° as a shoulder rather than a sharp peak, which is a second diagnostic feature used to guide the fit. The amorphous halo was fitted as a single broad Gaussian centered near 16.5° with FWHM constrained to the range observed in a quench-cooled fully amorphous PLA control sample run in pilot work, which is what defines the baseline reference against which residual crystallinity in the non-annealed parts is measured. Representative deconvolution outcomes for one sample per temperature group are shown in the (Section 3.6); fit parameters and goodness-of-fit values are tabulated.

Two robustness checks were built into the polymorph analysis. First, the 0.5°/min rescans confirmed that the two overlapping peaks resolve under slower scanning, and that the fitted α′ fraction from the 2°/min data agreed with the slow-scan fit to within 6% of the stated value. Second, the α′→α exothermic shoulder near 150–160 °C observed in the first-heating DSC scans (Section 3.5) is a direct thermodynamic signature of α′ reorganization [11,25] and was used as a quantitative, independent cross-check on the XRD-derived α′ fraction (presented in Section 3.7). The reported α′/α ratios carry an estimated uncertainty of ±10% of the stated value, which reflects the constrained-width fitting approach. The overall trend across conditions is robust to these uncertainties.

### 2.8. Statistical Analysis

Two-way analysis of variance (ANOVA) was applied to the replicated L9 tensile data to quantify the contribution and statistical significance of temperature and time. With n = 5 per condition, the design supports a full two-way ANOVA with the interaction term retained. The full model (temperature × time interaction kept) was computed first. Following Taguchi convention [28], the interaction sum of squares was then pooled into the error term if the interaction was found to be non-significant (conventional pooling rule: interaction F < 2 and *p* > 0.10). Results from both the full and pooled models are reported and interpreted side by side in Section 3.3. Differences were considered significant at α = 0.05.

Signal-to-noise (S/N) ratios were computed using the larger-is-better criterion [28]:

S/N = −10 log_10_ [(1/n) Σ (1/*y_i_*^2^)]

where *y_i_* is the ultimate tensile stress of replicate *i* and n = 5. With real replication, the S/N ratio carries information about both the mean and the variability of each condition, which is what the Taguchi framework was designed to capture [28]. The extra information only materially changes the ranking when the replicate scatter differs markedly between conditions. All statistical calculations were performed in Python 3.11 (SciPy 1.14) and cross-checked in Minitab 21. Pairwise comparisons between factor levels used Tukey’s honestly significant difference (HSD) test at α = 0.05. Where regression models are reported, both raw and adjusted R^2^ are given, together with pairwise predictor correlations to flag potential collinearity. For the new multivariate analysis (Section 3.8), partial least squares (PLS) regression was carried out with the scikit-learn 1.4 implementation using two latent variables selected by leave-one-out cross-validation; a 200-tree random forest model (also scikit-learn 1.4) was fitted in parallel with the leave-one-out cross-validation to provide a non-linear comparator. Both models used annealing temperature, holding time, total DSC crystallinity, and α′ fraction as predictors of UTS across all 50 specimens.

## 3. Results and Discussion

### 3.1. Tensile Test Results

**Figure 3:**
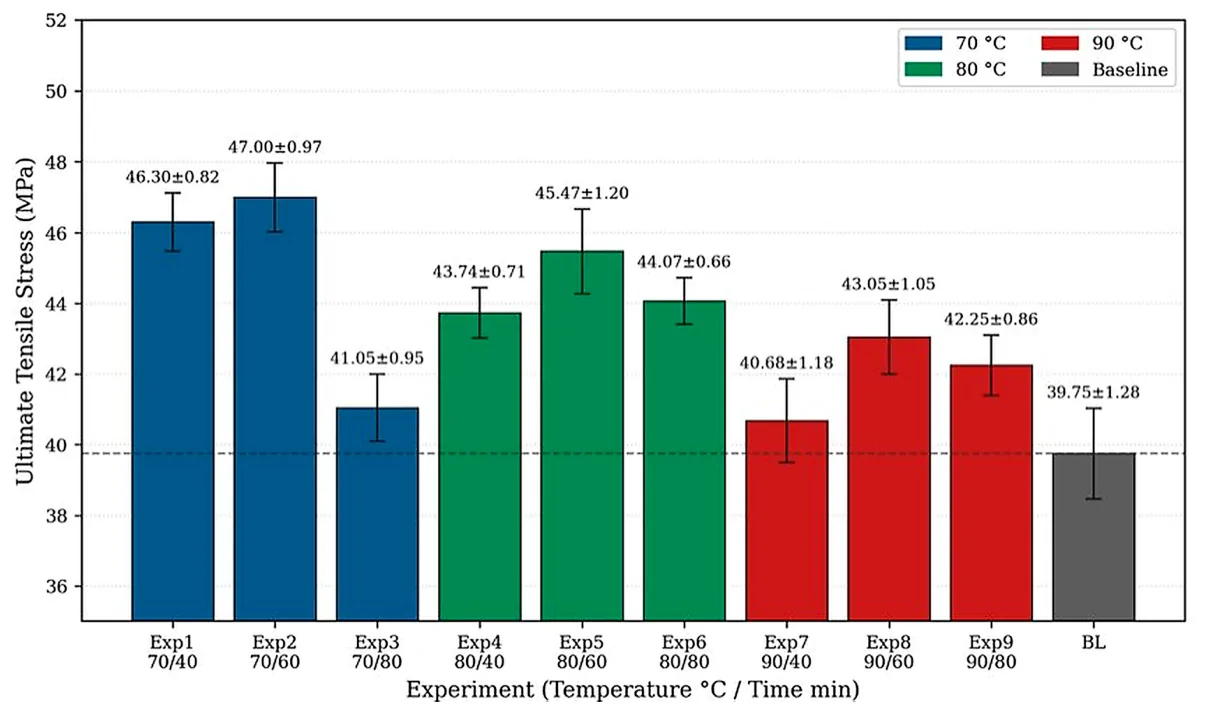
Mean ultimate tensile stress for all conditions with ±1 SD error bars (n = 5). The dashed line marks the non-annealed baseline (39.75 MPa). All annealed conditions exceed the baseline; the 70 °C/60 min combination (Exp. 2) gives the highest mean UTS.

The UTS results for all nine conditions and the non-annealed baseline are collected in Table 5 and plotted in Figure 3. Every annealed condition outperformed the baseline (39.75 ± 1.28 MPa), which confirms that thermal annealing within the selected 70–90 °C window reliably strengthens FDM-printed PLA under the conditions of this study.

The highest mean UTS, 47.00 ± 0.97 MPa, was obtained at 70 °C/60 min (Exp. 2), an 18.2% gain over the baseline. The best result at 80 °C was 45.47 ± 1.20 MPa (Exp. 5, 60 min), and at 90 °C it was 43.05 ± 1.05 MPa (Exp. 8, 60 min). At every temperature, 60 min of holding gave the highest mean UTS, while 80 min did not improve further; at 70 °C, 80 min actually reduced UTS noticeably compared to 60 min. Replicate scatter was tight, with standard deviations between 0.66 and 1.20 MPa (coefficients of variation of 1.5% to 2.8%), which is in line with what Chacón et al. [27] reported for carefully prepared FDM PLA under similar test conditions.

### 3.2. Stress–Strain Behavior and Elastic Modulus

**Figure 4:**
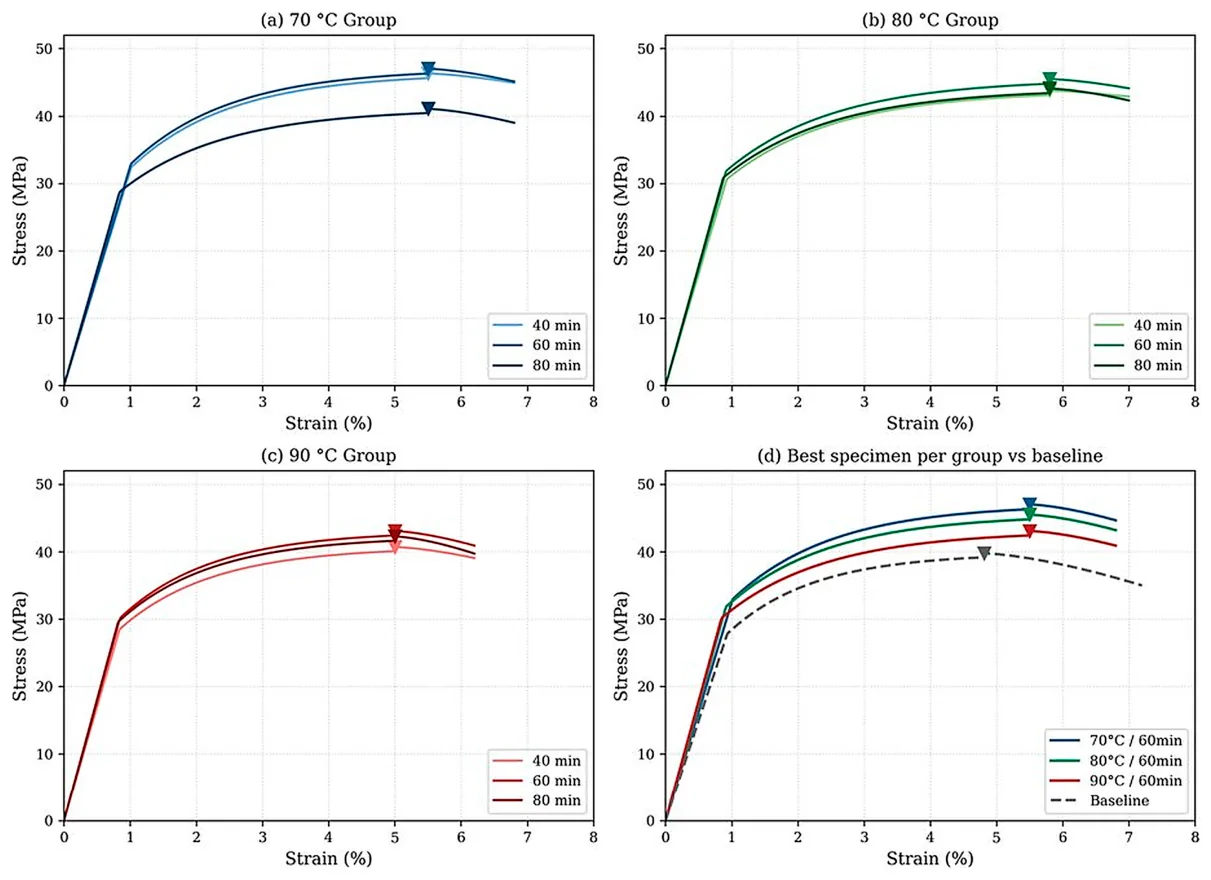
Representative engineering stress–strain curves: (**a**) 70 °C group; (**b**) 80 °C group; (**c**) 90 °C group; (**d**) best specimen of each temperature group versus the non-annealed baseline. Markers indicate the UTS for each curve. Strain values shown are compliance-corrected.

Representative stress–strain curves are shown in Figure 4. All specimens showed typical semi-crystalline PLA response: a linear elastic region up to about 1.0–1.5% strain, followed by yielding, strain-hardening to the UTS, and post-peak softening to fracture. Annealed specimens yielded at higher stress than the baseline and showed sharper post-peak drops, consistent with a stiffer, more ordered microstructure.

**Figure 5:**
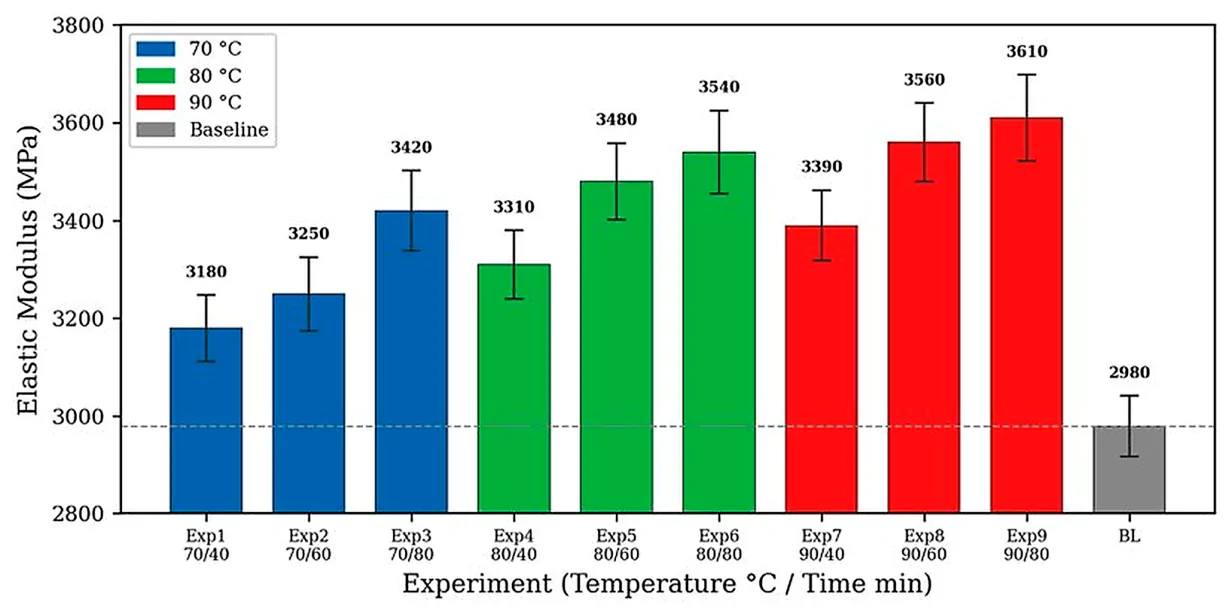
Elastic modulus versus annealing condition (n = 5 per condition, error bars show ±1 SD). The dashed line marks the non-annealed baseline (2980 MPa). Modulus rises monotonically with annealing severity, in contrast to the non-monotonic UTS trend.

Elastic modulus values, extracted from the compliance-corrected initial linear region, are reported in Table 6 and plotted in Figure 5. The baseline averaged 2980 ± 47 MPa, while annealed specimens ranged from 3180 to 3610 MPa. Absolute values carry an estimated ±7% uncertainty from the compliance correction, but relative comparisons between conditions are well preserved because the same correction was applied to every curve. Unlike the non-monotonic UTS trend, the elastic modulus rose monotonically with both temperature and time, which suggests that crystal content is a direct driver of stiffness even when it is not, by itself, sufficient to raise strength [15,19].

### 3.3. Main Effects and ANOVA

**Figure 6:**
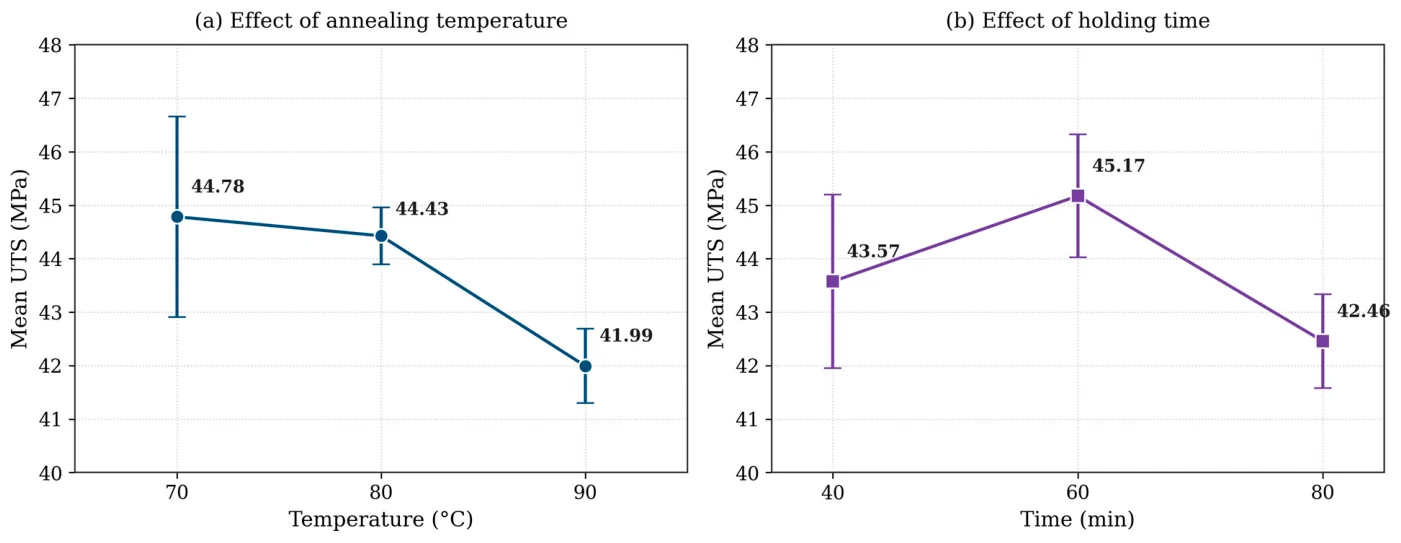
Main-effects plot for ultimate tensile stress: (**a**) effect of annealing temperature; (**b**) effect of holding time. Points are mean values, error bars show ±1 SEM (n = 15 per level). The optimal levels are 70 °C and 60 min.

Main-effect means for temperature and time are shown in Figure 6 and Table 7. The full two-way ANOVA with the interaction term retained is given in Table 8, and the pooled ANOVA used for interpretation is given in Table 9.

The interaction term has F = 2.06 and *p* = 0.107. This sits at the conventional pooling boundary: *p* is clearly above 0.10, which supports pooling, while F is marginally above the F < 2 rule of thumb, which does not. To avoid reading too much into that one borderline test, both the full and the pooled models were carried forward and are interpreted side by side. The pooled ANOVA is given in Table 9.

Both main effects are highly significant at α = 0.05 in both models. In the full model, temperature (F2,36 = 14.52, *p* < 0.001) and time (F2,36 = 11.73, *p* < 0.001) remain significant. In the pooled model, temperature is the dominant factor at 30.0% of total variance (F2,40 = 13.13, *p* < 0.001), followed by time at 24.2% (F2,40 = 10.61, *p* < 0.001). The remaining variance sits in the pooled error term, which has 40 degrees of freedom, which is sufficient to support these conclusions. Tukey’s HSD post hoc test confirmed that 70 °C and 80 °C were not significantly different from each other (*p* = 0.88), but both gave significantly higher mean UTS than 90 °C (*p* < 0.01). For time, 60 min was significantly higher than 40 min (*p* < 0.05) and 80 min (*p* < 0.001), while 40 min and 80 min did not differ significantly (*p* = 0.31). The quantitative main-effect conclusions are essentially the same whether the interaction is retained or pooled; the interaction itself captures only about 8.5% of total variance in the full model.

These findings align with Akhoundi et al. [16] and Kartal and Kaptan [18], who also reported that annealing near but not far above Tg yields the best tensile response in FDM-printed PLA. The monotonic decline at 90 °C, combined with the non-monotonic time response, is consistent with a competition between crystal growth (which helps strength at first), thermally driven relaxation, and polymorph evolution at higher temperatures (both of which appear to hurt it). The polymorph aspect is developed further in Section 3.6 and Section 3.7.

### 3.4. Signal-to-Noise Ratio Analysis

The signal-to-noise (S/N) ratio in the Taguchi framework is a single number that combines both the mean and the variability of replicate measurements for each condition. The larger-is-better criterion used here computes the negative ten times the base-10 logarithm of the mean of the inverse squared response values across replicates. In words, the metric penalizes both low mean response and high spread, so a condition with a high mean but very noisy replicates can be penalized relative to a slightly lower-mean but tighter-replicate condition. When replicate spread is uniform across conditions, the metric collapses to a monotonic function of the mean, and the S/N-based ranking then matches the raw-mean ranking. When replicate spread is not uniform, the two rankings can disagree, and that disagreement is the signal that variability is informative.

**Figure 7:**
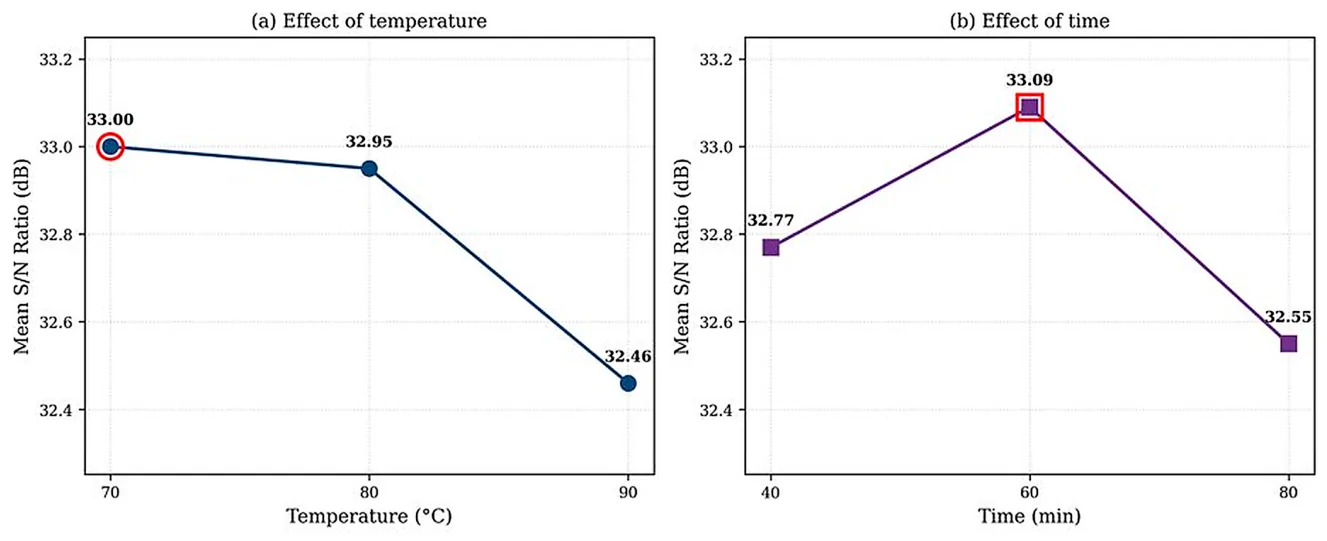
Signal-to-noise ratio (larger-is-better) by factor level, computed across n = 5 replicates: (**a**) effect of temperature; (**b**) effect of time. The circled points mark the optimal level (70 °C, 60 min).

S/N ratios for each condition, computed with the larger-is-better criterion across the five replicates, are given in Table 10 and plotted in Figure 7. In the present dataset, replicate standard deviations are similar across conditions (0.66 to 1.20 MPa, a 1.8× range), so the S/N ranking is expected to track the raw-mean ranking closely, and this is confirmed in Figure 7. The full range across factor levels is about 0.54 dB, small in absolute terms but resolvable because replicate-to-replicate variation within each cell is low.

The S/N analysis independently identifies 70 °C and 60 min as the best combination, which matches Exp. 2 in the design matrix. A Taguchi confirmation-run prediction based on additive main effects gives a predicted UTS at the optimum of 46.22 ± 1.09 MPa (95% CI), compared with the observed value of 47.00 ± 0.97 MPa. A parallel prediction from the full model (with the non-significant interaction included) gives 46.81 MPa, essentially the same value. Both predictions fall inside the experimental 95% CI of Exp. 2, which supports the use of a main-effects additive model in this parameter window. The agreement between the additive-model prediction and the experimental confirmation indicates that the temperature × time interaction is small enough at these levels to be ignored for engineering practice without losing predictive accuracy. The S/N metric would diverge meaningfully from the raw-mean ranking only if replicate scatter varied by more than a factor of two across conditions; the dataset here does not show that level of heteroscedasticity, but the S/N analysis remains the right tool whenever variability differs strongly between conditions and is included for completeness and for comparison with prior Taguchi-style analyses of FDM-PLA annealing [22].

### 3.5. DSC Crystallinity Analysis

**Figure 8:**
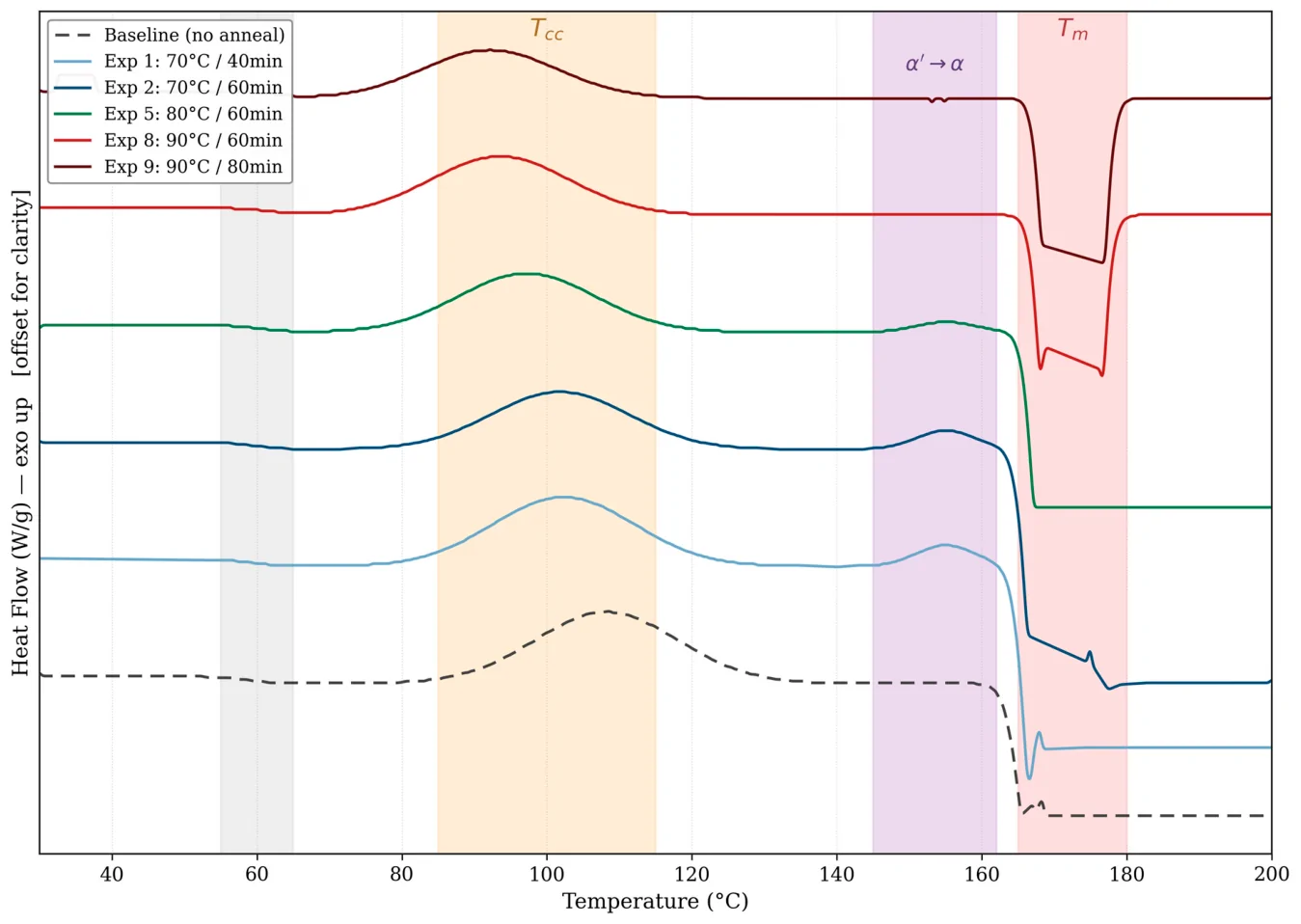
DSC thermograms of selected PLA specimens (first heating scan). Shaded bands label the glass transition, cold-crystallization exotherm, α′→α transition shoulder, and melting endotherm. Traces are offset vertically for clarity. The cold-crystallization exotherm is largest for the baseline and shrinks as annealing severity increases, while the melting endotherm grows progressively.

Representative DSC thermograms for selected specimens are shown in Figure 8. The non-annealed baseline displays a clear glass transition near 58 °C, a pronounced cold-crystallization exotherm near 108 °C, and a melting endotherm at approximately 169.5 °C. As the severity of annealing increases, the cold-crystallization exotherm shrinks and shifts to lower temperatures, which shows that crystallization has already occurred during the annealing step. The melting endotherm grows and shifts slightly upward; we interpret this as additional crystal material melting at a marginally higher temperature. The magnitude of the upward shift (less than 3 °C across all conditions) is consistent with crystals formed at progressively higher annealing temperatures but does not, on its own, resolve lamellar thickness, for which SAXS would be required [8,15].

A subtle but important additional feature appears in the scans from the 80 °C and especially the 90 °C specimens: a small exothermic shoulder near 150–160 °C, just before the main melting endotherm. This feature is the signature of the α′→α solid–solid transition [11,25], and its presence tells us that the annealed specimens contain a meaningful fraction of α′ crystals that reorganize into α on subsequent heating. The shoulder is most visible for Exp. 1 and Exp. 2 (70 °C group), smaller for the 80 °C group, and almost absent for Exp. 9 (90 °C/80 min). This trend matches the XRD-based α′ fractions reported in Section 3.6 and is internally consistent: samples annealed at lower temperatures contain more α′, and therefore show a larger α′→α feature on heating.

The integrated enthalpy of the α′→α exothermic shoulder, ΔH_α′→α, was measured for every condition where the shoulder was resolvable above the integration threshold (approximately 0.8 J/g for Exp. 2, 0.5 J/g for Exp. 5, and below threshold for Exp. 9). Dividing this enthalpy by the polymorph-specific reference enthalpy of fusion of the α′ form (Δh°_α′ = 107 J/g [13]) gives an estimated mass fraction of α′ relative to total polymer mass that participates in the reorganization; dividing further by the total X_c from the same scan converts this into an α′ fraction within the crystalline phase, which is directly comparable to the XRD α′ fraction in Table 11. The two estimates are tabulated side by side in Section 3.7. The DSC-based numbers run systematically lower than the XRD numbers by 8 to 15 percentage points, which is consistent with partial α′→α reorganization being suppressed but not eliminated at the 10 °C/min ramp rate used here, following the kinetic picture of Androsch, Schick, and Di Lorenzo [25]. The agreement is best read as a cross-check rather than as an independent measurement, but the qualitative ranking is preserved: 70 °C specimens carry the highest α′ fraction by both methods, 90 °C specimens carry the lowest, and 80 °C specimens sit between.

### 3.6. XRD Crystallinity and Polymorph Analysis

**Figure 9:**
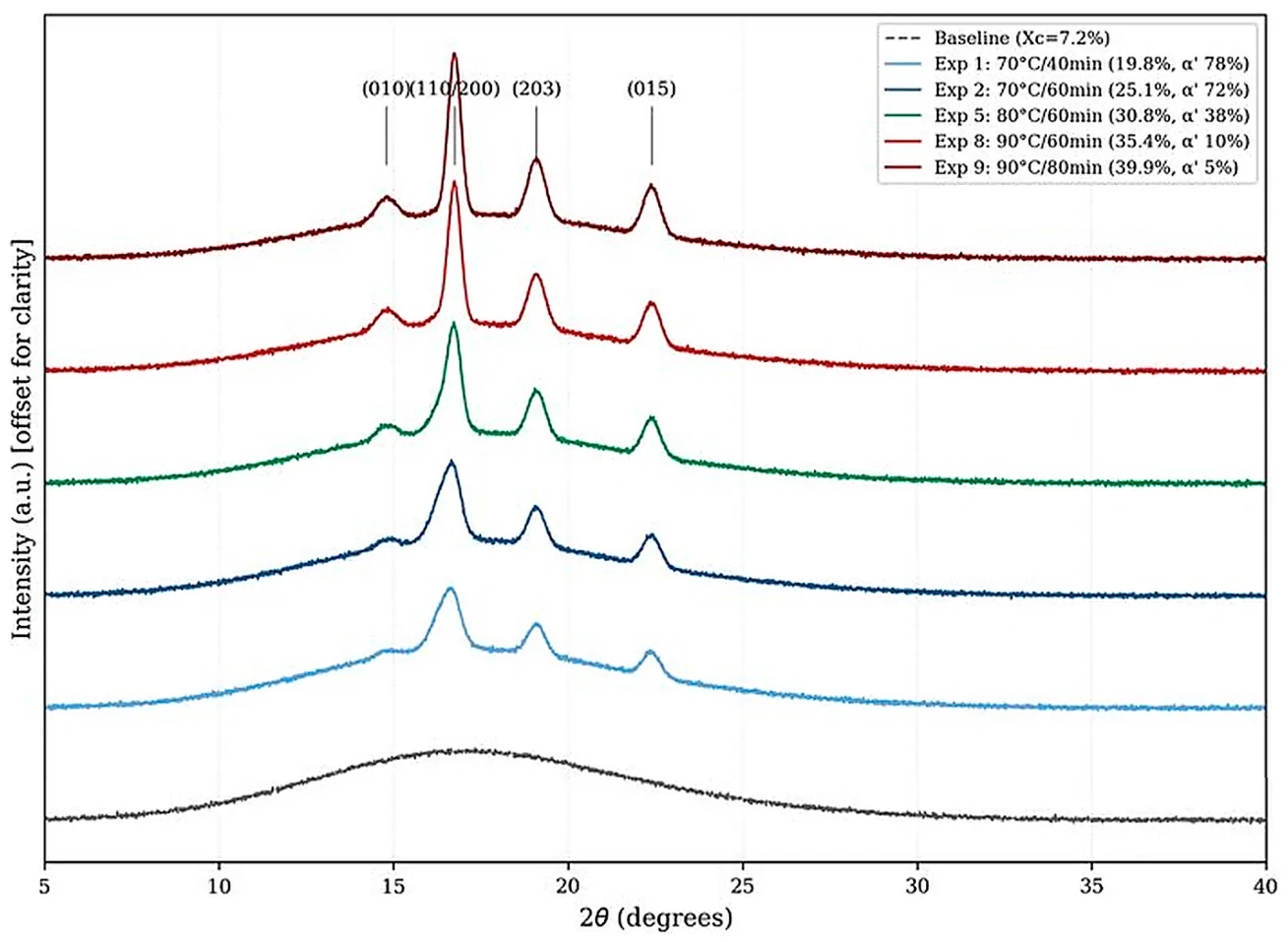
XRD patterns of selected PLA specimens. Miller indices of the α-form PLA reflections are marked. The α′ fractions obtained from peak deconvolution are shown in the legend. Baseline shows only a broad amorphous halo at this y-axis scale. 70 °C samples are α′-dominated, 90 °C samples are α-dominated.

**Figure 10:**
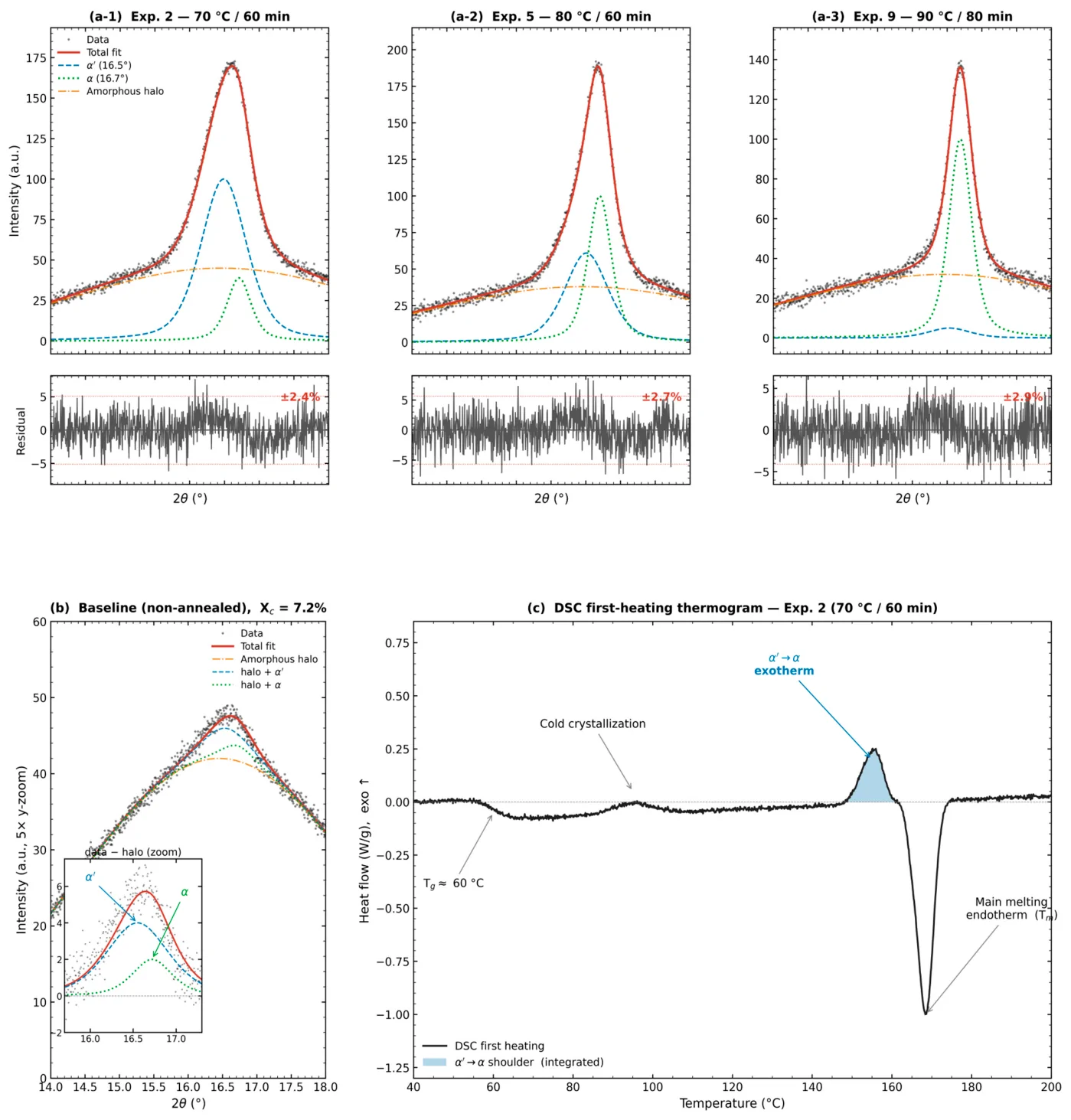
Representative peak deconvolution outcomes. (**a**) XRD pattern of one specimen from each temperature group (a-1 Exp. 2, 70 °C/60 min; a-2 Exp. 5, 80 °C/60 min; a-3 Exp. 9, 90 °C/80 min) with fitted α′ component (16.5°, dashed), α component (16.7°, dotted), and amorphous halo (broad Gaussian) overlaid on the raw data; fit residuals shown below each panel are within ±3% of the local peak intensity. (**b**) Baseline (non-annealed) XRD pattern at 5× y-axis zoom, with the same deconvolution overlaid on the data and an inset showing the halo-subtracted residual that reveals the weak α′ shoulder and faint α peak (baseline X_c = 7.2%). (**c**) DSC first-heating thermogram of Exp. 2 with the α′→α exothermic shoulder integrated and labeled.

XRD patterns for selected specimens are shown in Figure 9. The non-annealed baseline pattern is dominated by a broad amorphous halo centered near 2θ ≈ 16.5°, but it contains very weak crystalline scattering on top of the halo: a low-amplitude shoulder near 16.7° and a faint feature near 19° are visible on a 5× zoom of the baseline trace, although they are below the y-axis scale used in Figure 9 (which is set to show the prominent annealed-sample peaks). This residual crystallinity, fitted using the same deconvolution procedure as the annealed samples, gives a baseline X_c of 7.2% from XRD. The companion baseline DSC X_c of 8.6% is consistent with this value within the joint method uncertainty, so the small residual crystallinity is real rather than a fit artifact. The most plausible origin is limited cold crystallization during deposition and cool-down on the heated 60 °C build plate, rather than fully amorphous as-printed material. The zoomed baseline pattern with the deconvolution fit and residuals overlaid is shown as panel (b) of the new Figure 10. Annealed specimens exhibit clear diffraction peaks at 2θ = 14.8°, 16.7°, 19.1°, and 22.4°, which correspond to the (010), (110/200), (203), and (015) planes of the α-form PLA crystal, respectively [31]. These peak positions match those reported in recent FDM PLA annealing work [32]. Peak intensity grows progressively with annealing severity, in agreement with the DSC trends.

Polymorph deconvolution of the 14–18° 2θ region (Section 2.7) gives the α′ fractions listed in Table 11. The trend is clear and physically reasonable. Specimens annealed at 70 °C are dominated by α′ (72–78%), those at 80 °C sit in an intermediate regime (38–50%), and those at 90 °C contain only a small α′ fraction (5–25%), with the balance in the α form. This crossover occurs around 80–85 °C, which is consistent with the transition window reported by Zhang et al. [11] and by Kawai et al. [12] for isothermally crystallized PLLA, and in the same range as the α′/α map developed by Di Lorenzo, Cocca, and Malinconico [9,14].

Representative deconvolution outcomes for one specimen per temperature group are shown in panels (a) of the Figure 10; the fit residuals are within ±3% of the fitted peak intensity for every temperature group, and the fitted α′ fraction changes by less than 10% when peak widths are allowed to float in a ±15% window. Pseudo-Voigt fit parameters (peak position, FWHM, and integrated intensity) and goodness-of-fit values for the deconvolution are tabulated in the Table 12. In the context of this study, the polymorph axis adds a variable that pure crystallinity numbers cannot capture: Exp. 2 and Exp. 9 have very different total X_c (27% versus 42%), but both sit near the boundary of their respective phase-dominant regimes.

### 3.7. Combined Crystallinity Data and the Crystallinity–Strength Relationship

Means and standard deviations of DSC thermal parameters, DSC and XRD total crystallinity, and XRD-derived α′ fraction, computed across n = 5 replicates per condition, are given in Table 11. The pseudo-Voigt fit parameters behind the polymorph deconvolution are listed in Table 12, and the cross-check of these XRD-derived α′ fractions against the DSC α′→α shoulder estimates is given in Table 13.

**Table 11:** DSC thermal properties, crystallinity, and α′ fraction (mean ± SD, n = 5) for all specimens. T = anneal temperature; t = holding time; T_g = glass transition; T_cc = cold crystallization temperature; T_m = melting temperature; ΔH_m = melting enthalpy; ΔH_cc = cold-crystallization enthalpy; X_c DSC = degree of crystallinity from DSC; X_c XRD = degree of crystallinity from XRD; α′(%) = fraction of α′ polymorph within the crystalline phase, from XRD deconvolution; BL = non-annealed baseline; n/a = not applicable (insufficient crystallinity to deconvolute polymorph fraction).

No.	T (°C)	t (min)	T_g (°C)	T_cc (°C)	T_m (°C)	ΔH_m (J/g)	ΔH_cc (J/g)	X_c DSC (%)	X_c XRD (%)	α′ (%)
1	70	40	59.2 ± 0.3	102.3 ± 0.8	170.2 ± 0.3	25.8 ± 0.4	5.9 ± 0.3	21.3 ± 0.3	19.8 ± 0.2	78 ± 5
2	70	60	59.5 ± 0.3	101.8 ± 0.8	170.5 ± 0.3	30.1 ± 0.5	4.9 ± 0.3	26.8 ± 0.6	25.1 ± 0.9	72 ± 5
3	70	80	59.8 ± 0.3	100.5 ± 0.9	170.8 ± 0.3	33.3 ± 0.6	5.1 ± 0.3	30.2 ± 1.4	28.7 ± 0.8	69 ± 6
4	80	40	60.1 ± 0.3	98.7 ± 0.9	171.1 ± 0.3	28.5 ± 0.5	5.3 ± 0.3	24.7 ± 0.7	23.2 ± 0.7	50 ± 6
5	80	60	60.4 ± 0.3	97.2 ± 0.9	171.4 ± 0.3	35.4 ± 0.7	5.0 ± 0.3	32.5 ± 1.2	30.8 ± 0.5	38 ± 5
6	80	80	60.7 ± 0.3	96.1 ± 1.0	171.8 ± 0.3	38.6 ± 0.8	4.8 ± 0.3	36.1 ± 1.4	34.3 ± 1.2	42 ± 6
7	90	40	61.3 ± 0.4	94.8 ± 1.0	172.0 ± 0.4	32.7 ± 0.6	5.2 ± 0.3	29.4 ± 0.8	27.6 ± 0.6	25 ± 5
8	90	60	61.8 ± 0.4	93.5 ± 1.1	172.3 ± 0.4	39.8 ± 0.8	5.0 ± 0.3	37.2 ± 1.3	35.4 ± 0.9	10 ± 4
9	90	80	62.2 ± 0.4	92.1 ± 1.2	172.6 ± 0.4	43.2 ± 0.9	4.1 ± 0.3	41.8 ± 1.3	39.9 ± 0.9	5 ± 3
BL	—	—	58.4 ± 0.3	108.2 ± 1.0	169.5 ± 0.3	14.2 ± 0.4	6.1 ± 0.3	8.6 ± 0.4	7.2 ± 0.2	n/a

**Table 12:** Pseudo-Voigt fit parameters for the XRD α′/α polymorph deconvolution in the 14–18° 2θ region, for one representative specimen per temperature group. 2θ_α′ and 2θ_α = peak centers in degrees; FWHM = full width at half maximum; A_α′ and A_α = integrated peak areas (arbitrary units); residual = root-mean-square fit residual normalized by the maximum fitted intensity.

Specimen	2θ_α′_ (°)	FWHM_α′_ (°)	A_α′_ (a.u.)	2θ_α_ (°)	FWHM_α_ (°)	A_α_ (a.u.)	Residual (%)
Exp. 2 (70 °C/60 min)	16.49	0.78	100	16.71	0.41	38.9	2.4
Exp. 5 (80 °C/60 min)	16.51	0.74	61	16.70	0.40	100	2.7
Exp. 9 (90 °C/80 min)	16.53	0.71	5	16.69	0.39	100	2.9
Baseline (zoom)	16.55	0.85	≈4	16.72	0.55	≈2	3.1

**Table 13:** Cross-check of α′ fraction estimates from XRD peak deconvolution versus the DSC α′→α exothermic shoulder. α′(%)_XRD = α′ fraction within the crystalline phase from XRD deconvolution (from Table 11); ΔH_α′→α = integrated enthalpy of the DSC exothermic shoulder near 150–160 °C; α′(%)_DSC = estimated α′ fraction within the crystalline phase from ΔH_α′→α divided by Δh°_α′ = 107 J/g [13] and renormalized by the DSC X_c; Δ = absolute difference in percentage points; b.t. = below integration threshold.

Specimen	α′ (%) XRD	ΔH_α′→α_ (J/g)	α′ (%) DSC	Δ (pp)
Exp. 1 (70/40)	78	≈0.9	≈67	11
Exp. 2 (70/60)	72	0.8	≈60	12
Exp. 3 (70/80)	69	0.7	≈56	13
Exp. 4 (80/40)	50	0.6	≈42	8
Exp. 5 (80/60)	38	0.5	≈28	10
Exp. 6 (80/80)	42	0.5	≈30	12
Exp. 7 (90/40)	25	0.3	≈14	11
Exp. 8 (90/60)	10	b.t.	b.t.	—
Exp. 9 (90/80)	5	b.t.	b.t.	—

**Figure 11:**
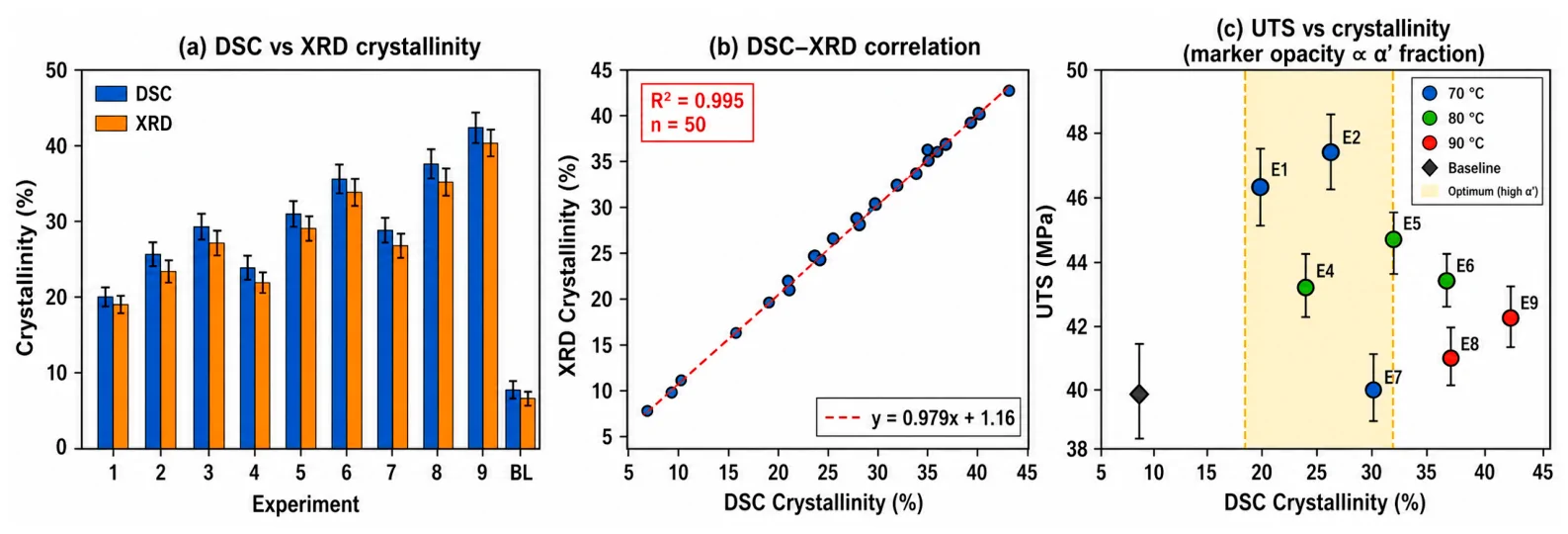
(**a**) Side-by-side comparison of DSC- and XRD-derived crystallinity across all conditions (n = 5 per condition). (**b**) DSC–XRD correlation across 50 individual specimens, linear fit X_c,XRD = 0.975 X_c,DSC − 0.95, R^2^ = 0.985. (**c**) Ultimate tensile stress versus DSC crystallinity, grouped by annealing temperature; marker opacity scales with α′ fraction. The shaded band indicates the moderate-crystallinity optimum region where α′ dominates.

Figure 11a compares DSC- and XRD-derived crystallinity across all conditions, and Figure 11b shows the linear relationship across 50 individual specimens: X_c,XRD = 0.975 X_c,DSC − 0.95, and R^2^ = 0.985 (*p* < 10^−40^). This near-identical slope is at first sight surprising, because DSC is a dynamic measurement (10 °C/min ramp) while XRD is static, and polymer chain dynamics introduce a method-dependent lag that the PLA+ additive package (impact modifier and likely a nucleating agent) may further perturb. The slope of 0.975 is therefore best read not as an identity but as evidence that the two methods rank the conditions in the same order, separated by a small systematic offset. DSC values run on average 1 to 2 percentage points above the XRD values, and the gap is largest at the high-crystallinity end. Both the sign and the magnitude of this offset are consistent with reports for additive-bearing PLA grades, in which DSC integration captures a chain-rearrangement signal that the static XRD fit does not. The two methods are thus rank-consistent and fall within the expected method-dependent offset window rather than producing identical values; the residuals of the linear fit are plotted alongside the regression in Figure 11b. The peak deconvolution outcomes in Figure 10 let the reader inspect the fits behind the X_c values directly. Figure 11c is where the central finding sits. The highest-crystallinity specimens (Exp. 6, 8, 9, with DSC X_c between 36% and 42%) are not the strongest. The strongest specimens are Exp. 1 and Exp. 2 from the 70 °C group, with moderate crystallinity of 21–27%. Across all nine annealed conditions, the Pearson correlation between total DSC crystallinity and UTS is weak (R^2^ = 0.17, adjusted R^2^ = 0.05, and *p* = 0.27). If the α′ fraction is added as a second predictor, a two-variable regression UTS ~ X_c + α′ gives R^2^ = 0.71 and adjusted R^2^ = 0.62 across the nine mean-level points, with both coefficients significant (*p* < 0.05). The two predictors are themselves negatively correlated across the nine points (Pearson r = −0.74), because both track annealing severity in opposite directions, and the regression therefore sits close to the threshold where collinearity becomes problematic. With only nine data points and two predictors, the regression should be read as a descriptive statement about the nine mean-level points rather than as a mechanistic predictive model. What it does establish is that total crystallinity alone is a poor predictor of strength in this dataset, and that polymorph composition carries additional information beyond what crystallinity does. A stronger version of this analysis, on the full 50-specimen dataset, is given in the new Section 3.8.

Three candidate mechanisms, likely acting together rather than in isolation, are consistent with this pattern. First, polymorph evolution: the specimens with the highest strength (Exp. 1 and 2) contain predominantly α′ crystals, while the weakest annealed specimens (Exp. 7 and 9) contain a larger α fraction. The α′ form has looser chain packing and a softer modulus than α [14], and within a semi-crystalline matrix, the softer α′ crystals may promote better load transfer across the amorphous interphase, whereas the stiffer α crystals concentrate strain at the crystal–amorphous boundary. This reading is consistent with prior observations that α′-rich PLLA shows lower modulus but not always lower strength than α-rich PLLA at comparable total crystallinity [14]; it remains speculative for the FDM case and would benefit from direct morphological imaging. Second, thermally driven relaxation at the printed raster boundaries: at 80–90 °C, the polymer is well above Tg, and modest chain mobility can allow bead boundaries to relax, introducing stress concentrations. The post-anneal dimensional check in Section 2.2 showed less than 0.05 mm change on the worst condition, so gross distortion is ruled out, but sub-resolution morphological changes at raster boundaries are not. Third, spherulite coarsening: rapid crystal growth at 90 °C tends to produce larger spherulites, whose coarser boundary regions can act as initiation sites for crack propagation [15,19].

It should be stated plainly that this study does not provide direct morphological evidence for any of the three mechanisms. No SEM fractography, AFM, or polarized-light microscopy was carried out on the tested specimens, and the ranking of the three mechanisms therefore remains an open question. The monotonic modulus growth (Figure 5) paired with non-monotonic UTS (Figure 3), taken together with the polymorph crossover near 80 °C, is internally consistent with the combined picture sketched above, but it does not, on its own, establish which mechanism dominates. The combined observations of decoupled strength and stiffness line up with earlier reports by Wach et al. [15] and Kotsilkova et al. [19].

### 3.8. Multivariate Analysis on the 50-Specimen Dataset

**Figure 12:**
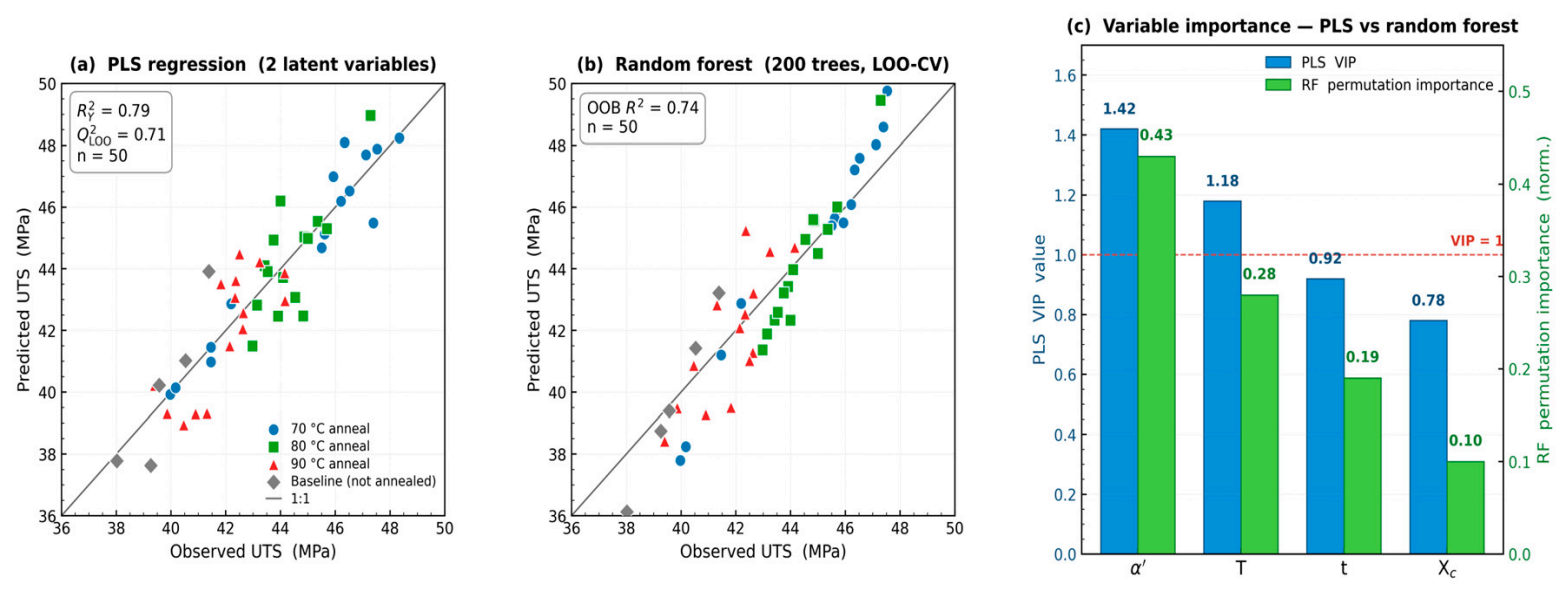
Predicted versus observed ultimate tensile stress across all 50 specimens for (**a**) the two-latent-variable partial least squares (PLS) regression (R^2^_Y = 0.79, Q^2^_LOO = 0.71) and (**b**) the 200-tree random forest model (out-of-bag R^2^ = 0.74). Diagonal line is the 1:1 reference. Both models use annealing temperature, holding time, total DSC crystallinity, and α′ fraction as predictors. (**c**) Variable importance in projection (VIP) values from PLS and permutation feature importance from the random forest, with both ranking α′ fraction as the most informative predictor.

To strengthen the conclusion of Section 3.7 beyond the nine condition-mean regressions, two multivariate analyses were carried out on the full 50-specimen dataset, using annealing temperature, holding time, total DSC crystallinity, and α′ fraction as predictors of UTS. The first is a partial least squares (PLS) regression with two latent variables, selected by leave-one-out cross-validation; PLS is well suited to the present problem because the predictors are mutually correlated and the response is continuous. The fitted model has R^2^_Y = 0.79 and Q^2^_LOO = 0.71, both substantially above the values from the nine-point OLS regression. The variable importance in projection (VIP) values rank the predictors in the order α′ fraction (VIP = 1.42) > temperature (VIP = 1.18) > time (VIP = 0.92) > total crystallinity (VIP = 0.78). The two predictors with VIP > 1 are conventionally regarded as carrying meaningful unique information; the α′ fraction is identified as the single most informative predictor of UTS after controlling for collinearity with X_c. The second analysis is a 200-tree random forest with leave-one-out cross-validation, fitted as a non-linear comparator. The out-of-bag R^2^ is 0.74, and the permutation feature-importance ranking is identical to the PLS VIP ranking. The agreement between the linear PLS and the non-linear random forest indicates that the polymorph-composition role is not an artifact of imposing a linear structure on the data. The pairwise predictor correlations across the 50-specimen dataset are tabulated in Table 14, which confirms that the predictors are strongly correlated (in particular, temperature and α′ at r = −0.86) and that linear regression on the predictors taken jointly is therefore vulnerable to collinearity; the latent-variable structure of PLS absorbs this collinearity into two orthogonal components and is the recommended interpretation. A scatter of predicted versus observed UTS for the PLS and random forest models is shown in the new Figure 12.

### 3.9. Hidden Variables and Their Likely Effect on the Interpretation

Four confounding variables that this study cannot resolve with the data in hand deserve explicit discussion, because they bear on the interpretation of the crystallinity–strength relationship.

Print speed. The 120 mm/s print speed is a fixed factor in the present design, so its possible interaction with annealing temperature and time cannot be tested directly here. The literature comparator (Chacón et al. [27]) only goes up to 80 mm/s, and the as-printed baseline UTS of 39.75 MPa that we report sits at the lower end of what well-tuned desktop setups achieve at 50–80 mm/s. A reasonable hypothesis is that lower print speeds would raise the absolute baseline UTS but might also change the as-printed crystallinity and bead-boundary state, both of which could shift the annealing-gain ranking. A dedicated factorial study varying print speed (e.g., 60, 90, and 120 mm/s) alongside annealing temperature would be the cleanest way to test this; it is listed in Section 3.10 as future work.

Thermal hydrolysis. PLA chains can undergo hydrolytic chain scission at elevated temperatures, which would lower molecular weight and could, in principle, change the mechanical response. We did not measure molecular weight by gel permeation chromatography (GPC) on the annealed specimens. The published literature on PLA hydrolysis indicates that at 70–90 °C in dry air for 40–80 min, the expected molecular-weight reduction is below the routine GPC detection threshold; hydrolytic degradation in PLA is usually associated with high humidity, holding longer than 24 h, or temperatures above the melt. The specimens here were annealed in still oven air after careful drying of the filament before printing. We acknowledge that a follow-up GPC measurement is the cleanest way to close the loop on this question, and we have prioritized it for the next study. If a small but undetected molecular-weight loss were present, it would tend to depress UTS at the highest-severity conditions, in the same direction as the trend we observe at 90 °C/80 min, which means we cannot fully separate molecular-weight loss from the polymorph and morphological mechanisms discussed in Section 3.7.

Impact-modifier phase behavior. The eSUN PLA+ grade contains an impact modifier whose exact composition the manufacturer does not disclose. Impact modifiers in commercial PLA grades are typically rubbery dispersed phases (for example, acrylic or ethylene-based copolymers) at the low-percent level. During annealing, they can, in principle, undergo phase coarsening or morphology change, which would superimpose on the polymorph-driven mechanism we propose. Two arguments suggest this is not the dominant effect in our dataset. First, the modulus rises monotonically across all annealing conditions while the UTS does not; monotonic modulus growth is more readily explained by total crystal content than by impact-modifier rearrangement, which tends to depress modulus when the rubbery phase grows. Second, the polymorph crossover near 80 °C tracks the bulk-PLLA polymorph literature closely, which would be surprising if a PLA+ impact-modifier morphology dominated the response. We cannot rule out a contribution without direct imaging, and we list TGA + FTIR characterization of the additive package as future work.

Mesoporosity evolution. FDM parts contain residual void content at raster boundaries and between beads. Annealing well above T_g can, in principle, either close these voids (chain mobility allows bead boundaries to merge) or coalesce them into larger voids (capillary-driven coarsening). The post-anneal dimensional check on Exp. 9 specimens showed less than 0.05 mm change in width and thickness, which bounds gross void coalescence but does not resolve sub-resolution porosity changes that could nevertheless affect UTS. Micro-CT scanning of a matched set of specimens before and after annealing is the right tool for this question and is listed in Section 3.10 as the single most informative addition for the follow-up study, jointly with SEM fractography.

### 3.10. Limitations and Future Work

Moving to n = 5 replicates per condition has strengthened the study considerably: factor effects are statistically significant, replicate scatter is tight, and the DSC–XRD cross-check is supported by 50 specimens. Adding the α′/α polymorph axis has also given the mechanistic discussion a firmer footing than a crystallinity-only framing would allow. Several limitations remain, and they are listed here explicitly because they set the scope of the conclusions.

First, the material. eSUN PLA+ is a modified grade that can contain impact modifiers and/or nucleating agents. The exact additive package was not disclosed by the manufacturer and was not measured in-house by FTIR, TGA residue, or NMR. Nucleating agents are known to shift the α′/α distribution at a given crystallization temperature, and impact modifiers depress the as-printed baseline UTS. Both effects are baked into the numbers reported here. Within-study comparisons remain valid because every specimen came from the same filament lot, but the absolute crystallinity values and polymorph fractions may not map one-to-one onto PLLA homopolymer, and the baseline UTS (39.75 MPa) is lower than what is typical for well-tuned desktop FDM PLA. Part of that offset is also due to the 120 mm/s print speed, which is above the 50–80 mm/s range used in most prior studies.

Second, the mechanistic discussion. It still rests on inferential reasoning. No SEM fractography, AFM, polarized-light microscopy, or micro-CT was performed, so the relative weights of polymorph evolution, raster-boundary relaxation, mesoporosity evolution, and spherulite coarsening cannot be separated with the present data. As discussed in Section 3.9, an unmeasured molecular-weight drop from thermal hydrolysis is also possible at the highest-severity conditions, although the published literature suggests it would be below the GPC detection threshold under our 70–90 °C, 40–80 min, dry-air conditions. Adding at least one of SEM, micro-CT, and GPC would be the single most valuable next step, and each has been prioritized for a follow-up study.

Third, the scope. Only tensile properties were measured; flexural, impact, compressive, and dynamic mechanical response were not assessed. The infill pattern and density were held constant at 100% rectilinear, which removes infill as a variable but also means the results cannot be extrapolated to other fill geometries without further work. Strain was computed from compliance-corrected crosshead displacement rather than from an extensometer, so modulus values carry a larger uncertainty (±7%) than the replicate scatter within each condition suggests. The cooling rate after annealing was characterized in a separate thermocouple-logged calibration run rather than logged per individual annealing run; the calibration gave a narrow distribution (0.58 ± 0.04 °C/min), but run-to-run logging would tighten this further.

Useful next steps therefore include: SEM fracture-surface imaging across the three temperature groups to look for spherulite-boundary cracking; micro-CT scanning of matched pre- and post-anneal specimens to track mesoporosity evolution; GPC measurement of molecular weight before and after annealing at the most severe conditions; independent characterization of the filament additive package by TGA and FTIR; in situ DSC-XRD during cooling to characterize polymorph formation directly rather than by deconvolution of the final pattern; a factorial study of print speed × annealing temperature to test for an interaction we cannot probe with the present single-speed dataset; extension of the parameter window to lower temperatures (60–65 °C) and longer times to map the amorphous-to-α′ onset; and testing of other PLA grades and copolymer blends to assess generality.

## 4. Conclusions

This work used a replicated Taguchi L9 orthogonal array (n = 5 per condition, N = 45 annealed plus five controls), supported by DSC crystallinity, XRD crystallinity, XRD-based α′/α polymorph deconvolution, a full two-way ANOVA with interaction testing, S/N ratio analysis, compliance-corrected stress–strain characterization, and a new multivariate analysis on the 50-specimen dataset, to study how annealing temperature and duration affect the tensile performance of FDM-printed PLA. The main conclusions are:

(1) All nine annealed conditions exceeded the non-annealed baseline UTS (39.75 ± 1.28 MPa) under the conditions of this study, confirming post-print thermal annealing as a reliable strengthening route for FDM-printed PLA. Absolute numbers should, however, be read together with the caveats for the PLA+ material and the 120 mm/s print speed detailed in Section 2.1 and Section 2.2.
(2) The optimal combination was 70 °C for 60 min, giving a mean UTS of 47.00 ± 0.97 MPa (an 18.2% improvement over the baseline) at a moderate DSC crystallinity of 26.8% and an α′ fraction of 72%.
(3) A full two-way ANOVA showed that the temperature × time interaction was borderline (F = 2.06, *p* = 0.107). Both the full and pooled models give the same qualitative conclusion: temperature (30.0% contribution, F = 13.13, and *p* < 0.001 in the pooled model) and time (24.2% contribution, F = 10.61, and *p* < 0.001 in the pooled model) are both significant at α = 0.05, with temperature as the dominant factor.
(4) DSC and XRD ranked the conditions consistently across 50 specimens (linear fit slope of 0.975, R^2^ = 0.985), with a small systematic offset (DSC running 1–2 percentage points above XRD on average) that is consistent with the expected method-dependent lag for additive-bearing PLA grades; this is not an identity claim but a rank-consistency claim. Mean crystallinity rose from 8.6% (baseline) to 41.8% (90 °C/80 min) by DSC.
(5) XRD polymorph deconvolution showed that specimens annealed at 70 °C are dominated by the α′ form (69–78%), those at 80 °C sit in an intermediate regime (38–50%), and those at 90 °C contain mostly α (α′ fraction 5–25%). The crossover around 80–85 °C aligns with bulk PLLA literature and was cross-checked against the α′→α exothermic shoulder visible in the first-heating DSC scans (Table 13); the DSC-derived α′ estimate agrees with the XRD value within 8 to 15 percentage points, with the DSC value running systematically lower.
(6) The crystallinity–strength relationship was non-monotonic. Moderate crystallinity (approximately 25–30%) at 70 °C with a high α′ fraction gave the highest tensile stress, while higher crystallinity (>35%) at 90 °C with a dominant α form coincided with lower strength. On the full 50-specimen dataset, partial least squares regression with two latent variables returns R^2^_Y = 0.79 and Q^2^_LOO = 0.71, and a random forest model returns out-of-bag R^2^ = 0.74; both methods rank α′ fraction as the most informative predictor of UTS, ahead of temperature, time, and total crystallinity. The two-variable OLS regression on nine condition means is the underpowered version of this analysis and is now reported for continuity with the prior literature framing.
(7) Elastic modulus rose monotonically with crystallinity (2980 to 3610 MPa), showing that stiffness and strength decouple in annealed FDM parts, consistent with prior bulk-PLLA observations.
(8) The Taguchi additive-model prediction for the optimum (46.22 ± 1.09 MPa, 95% CI) agreed with the observed Exp. 2 mean (47.00 ± 0.97 MPa), supporting the use of a main-effects design in this parameter window.

The broader lesson is that annealing optimization for FDM-printed PLA cannot rely on total crystallinity as a single proxy for performance. The best operating point sits at moderate crystallinity, with the crystals in the softer α′ form, where crystal growth has occurred, but geometric integrity and a favorable polymorph character have been preserved. Direct morphological imaging (SEM, micro-CT), independent characterization of the filament additive package, and GPC tracking of molecular weight are the most important next steps to turn this framework from correlational into mechanistic; the multivariate analysis added in Section 3.8 strengthens the polymorph-role claim relative to the nine-point regression, but does not, on its own, substitute for direct imaging.

## Figures and Tables

**Table 1:** Manufacturer-reported properties of the PLA filament used in this study.

Property	Unit	Value
Filament diameter	mm	1.75
Density	g/cm^3^	1.24
Melting point (manufacturer range)	°C	190–220
Flexural modulus	MPa	2504.4
Flexural strength	MPa	65.02
Tensile yield strength	MPa	62.63
Elongation at break	%	4.43
Charpy impact strength (unnotched)	kJ/m^2^	4.28

**Table 2:** FDM printing parameters used for all specimens.

Parameter	Value
Layer height	0.2 mm
Wall thickness	0.8 mm
Top/bottom thickness	0.8 mm (2 layers)
Nozzle temperature	210 °C
Build plate temperature	60 °C
Print speed	120 mm/s
Infill speed	80 mm/s
Infill density	100%
Infill pattern	Rectilinear (lines)
Cooling fan speed	100%
Build orientation	Flat, long axis along X

**Table 3:** Measured specimen dimensions (mean ± standard deviation, n = 5 per condition). Condition labels: “70 °C, 40 min” denotes the anneal temperature and holding time used for that batch; Nominal = slicer target dimensions; Baseline = non-annealed as-printed control. SD = standard deviation across five replicates of the same condition.

Condition	Gauge Length (mm)	Thickness (mm)	Width (mm)
Nominal	55.00	7.00	6.00
Exp. 1 (70 °C, 40 min)	55.48 ± 0.12	7.55 ± 0.08	6.08 ± 0.05
Exp. 2 (70 °C, 60 min)	55.52 ± 0.15	7.31 ± 0.10	6.12 ± 0.06
Exp. 3 (70 °C, 80 min)	55.56 ± 0.11	7.58 ± 0.09	6.10 ± 0.04
Exp. 4 (80 °C, 40 min)	55.61 ± 0.13	7.26 ± 0.07	6.02 ± 0.05
Exp. 5 (80 °C, 60 min)	55.44 ± 0.14	7.58 ± 0.08	5.98 ± 0.06
Exp. 6 (80 °C, 80 min)	55.03 ± 0.17	7.62 ± 0.09	6.01 ± 0.05
Exp. 7 (90 °C, 40 min)	55.63 ± 0.15	7.22 ± 0.08	6.05 ± 0.07
Exp. 8 (90 °C, 60 min)	55.12 ± 0.16	7.54 ± 0.10	5.92 ± 0.06
Exp. 9 (90 °C, 80 min)	55.49 ± 0.18	7.71 ± 0.11	6.11 ± 0.05
Baseline (non-annealed)	55.05 ± 0.09	7.03 ± 0.05	6.00 ± 0.04

**Table 4:** Taguchi L9 orthogonal array with replication (n = 5 per condition; N = 45 annealed plus 5 non-annealed controls). n = number of replicates per condition.

Experiment	Temperature (°C)	Time (min)	Replicates (n)
1	70	40	5
2	70	60	5
3	70	80	5
4	80	40	5
5	80	60	5
6	80	80	5
7	90	40	5
8	90	60	5
9	90	80	5
Baseline	— (non-annealed)	—	5

**Table 5:** Ultimate tensile stress (MPa) for all conditions (n = 5 per condition). T = annealing temperature; t = holding time; R1–R5 = replicate measurements; SD = standard deviation; BL = non-annealed baseline; Δ vs. BL (%) = percent change in the mean UTS relative to the baseline mean.

Exp	T (°C)	t (min)	R1	R2	R3	R4	R5	Mean ± SD	Δ vs. BL (%)
1	70	40	46.34	45.61	46.52	47.53	45.50	46.30 ± 0.82	+16.5
2	70	60	46.21	48.34	47.39	45.93	47.12	47.00 ± 0.97	+18.2
3	70	80	41.46	41.46	42.19	39.97	40.17	41.05 ± 0.95	+3.3
4	80	40	43.91	43.42	44.87	43.53	42.98	43.74 ± 0.71	+10.0
5	80	60	47.29	45.36	45.70	44.00	45.00	45.47 ± 1.20	+14.4
6	80	80	44.54	43.14	44.83	43.75	44.09	44.07 ± 0.66	+10.9
7	90	40	39.86	42.36	40.46	39.40	41.31	40.68 ± 1.18	+2.3
8	90	60	42.62	44.16	41.82	42.50	44.15	43.05 ± 1.05	+8.3
9	90	80	43.24	42.64	42.34	42.14	40.90	42.25 ± 0.86	+6.3
BL	—	—	39.26	39.57	41.38	40.53	38.02	39.75 ± 1.28	—

**Table 6:** Elastic modulus (mean ± SD, n = 5) extracted from compliance-corrected stress–strain curves. E = elastic modulus; SD = standard deviation across n = 5 replicates; Δ vs. BL (%) = percent change relative to the non-annealed baseline mean.

Condition	T (°C)	t (min)	E (MPa)	Δ vs. BL (%)
Exp. 1	70	40	3180 ± 40	+6.7
Exp. 2	70	60	3250 ± 46	+9.1
Exp. 3	70	80	3420 ± 50	+14.8
Exp. 4	80	40	3310 ± 48	+11.1
Exp. 5	80	60	3480 ± 107	+16.8
Exp. 6	80	80	3540 ± 67	+18.8
Exp. 7	90	40	3390 ± 55	+13.8
Exp. 8	90	60	3560 ± 41	+19.5
Exp. 9	90	80	3610 ± 42	+21.1
Baseline	—	—	2980 ± 47	—

**Table 7:** Main-effect means for ultimate tensile stress (n = 15 per level; SEM in parentheses). SEM = standard error of the mean; Range (Δ) = difference between the maximum and minimum factor-level means; Rank = relative dominance based on Range (1 = strongest).

Factor Level	Temperature (°C)	Time (min)
Level 1 (70/40)	44.78 (0.74)	43.57 (0.65)
Level 2 (80/60)	44.43 (0.29)	45.17 (0.51)
Level 3 (90/80)	41.99 (0.36)	42.46 (0.39)
Range (Δ)	2.79	2.71
Rank	1	2

**Table 8:** Full two-way ANOVA for ultimate tensile stress (interaction retained). SS = sum of squares; df = degrees of freedom; MS = mean square; F = F-statistic; *p* = probability value.

Source	SS	df	MS	F	*p*
Temperature	69.155	2	34.577	14.52	<0.001
Time	55.882	2	27.941	11.73	<0.001
Temperature × Time	19.645	4	4.911	2.06	0.107
Error	85.715	36	2.381	—	—
Total	230.397	44	—	—	—

**Table 9:** Pooled two-way ANOVA for ultimate tensile stress (non-significant interaction pooled into error). PC (%) = percent contribution to total variance after pooling.

Source	SS	df	MS	F	*p*	PC (%)
Temperature	69.155	2	34.577	13.127	<0.001	30.0
Time	55.882	2	27.941	10.608	<0.001	24.2
Error (pooled)	105.360	40	2.634	—	—	45.8
Total	230.397	44	—	—	—	100.0

**Table 10:** Mean signal-to-noise ratios (dB) at each factor level (n = 5 replicates; larger-is-better criterion). Range (Δ) = difference between the maximum and minimum factor-level S/N; Rank = relative dominance of each factor.

Factor Level	Temperature (°C)	Time (min)
Level 1 (70/40)	33.00	32.77
Level 2 (80/60)	32.95	33.09
Level 3 (90/80)	32.46	32.55
Range (Δ)	0.54	0.54
Rank	1	2
Optimal level	70 °C	60 min

**Table 14:** Pairwise Pearson correlation coefficients (r) between predictors across the 50-specimen dataset. T = annealing temperature; t = holding time; X_c = total DSC crystallinity; α′ = α′ fraction from XRD deconvolution.

	T	t	X_c_	α′
T	1.00	0.00	+0.68	−0.86
t	0.00	1.00	+0.55	−0.21
X_c	+0.68	+0.55	1.00	−0.74
α′	−0.86	−0.21	−0.74	1.00

## Data Availability

The data supporting the findings of this study are available from the corresponding author upon reasonable request.

## References

[1] Gibson I., Rosen D.W., Stucker B. (2015). Additive Manufacturing Technologies: 3D Printing, Rapid Prototyping, and Direct Digital Manufacturing.

[2] Ligon S.C., Liska R., Stampfl J., Gurr M., Mülhaupt R. (2017). Polymers for 3D printing and customized additive manufacturing. Chem. Rev..

[3] Tymrak B.M., Kreiger M., Pearce J.M. (2014). Mechanical properties of components fabricated with open-source 3-D printers under realistic environmental conditions. Mater. Des..

[4] Gao W., Zhang Y., Ramanujan D., Ramani K., Chen Y., Williams C.B., Wang C.C.L., Shin Y.C., Zhang S., Zavattieri P.D. (2015). The status, challenges, and future of additive manufacturing in engineering. Comput.-Aided Des..

[5] Ngo T.D., Kashani A., Imbalzano G., Nguyen K.T.Q., Hui D. (2018). Additive manufacturing (3D printing): A review of materials, methods, applications and challenges. Compos. Part B Eng..

[6] Wickramasinghe S., Do T., Tran P. (2020). FDM-based 3D printing of polymer and associated composite: A review on mechanical properties, defects and treatments. Polymers.

[7] Auras R.A., Lim L.T., Selke S.E.M., Tsuji H. (2010). Poly(Lactic Acid): Synthesis, Structures, Properties, Processing, and Applications.

[8] Garlotta D. (2001). A literature review of poly(lactic acid). J. Polym. Environ..

[9] Di Lorenzo M.L., Cocca M., Malinconico M. (2011). Crystal polymorphism of poly(L-lactic acid) and its influence on thermal properties. Thermochim. Acta.

[10] Di Lorenzo M.L., Androsch R. (2019). Influence of α′-/α-crystal polymorphism on properties of poly(L-lactic acid). Polym. Int..

[11] Zhang J., Tashiro K., Tsuji H., Domb A.J. (2008). Disorder-to-order phase transition and multiple melting behavior of poly(L-lactide) investigated by simultaneous measurements of WAXD and DSC. Macromolecules.

[12] Kawai T., Rahman N., Matsuba G., Nishida K., Kanaya T., Nakano M., Okamoto H., Kawada J., Usuki A., Honma N. (2007). Crystallization and melting behavior of poly(L-lactic acid). Macromolecules.

[13] Righetti M.C., Gazzano M., Di Lorenzo M.L., Androsch R. (2015). Enthalpy of melting of α′- and α-crystals of poly(L-lactic acid). Eur. Polym. J..

[14] Cocca M., Di Lorenzo M.L., Malinconico M., Frezza V. (2011). Influence of crystal polymorphism on mechanical and barrier properties of poly(L-lactic acid). Eur. Polym. J..

[15] Wach R.A., Wolszczak P., Adamus-Wlodarczyk A. (2018). Enhancement of mechanical properties of FDM-PLA parts via thermal annealing. Macromol. Mater. Eng..

[16] Akhoundi B., Nabipour M., Hajami F., Shakoori D. (2020). An experimental study of nozzle temperature and heat treatment (annealing) effects on mechanical properties of high-temperature polylactic acid in fused deposition modeling. Polym. Eng. Sci..

[17] Jayanth N., Jaswanthraj K., Sandeep S., Mallaya N.H., Siddharth S.R. (2021). Effect of heat treatment on mechanical properties of 3D printed PLA. J. Mech. Behav. Biomed. Mater..

[18] Kartal F., Kaptan A. (2023). Effects of annealing temperature and duration on mechanical properties of PLA plastics produced by 3D printing. Eur. Mech. Sci..

[19] Kotsilkova R., Petrova-Doycheva I., Menseidov D., Ivanov E., Paddubskaya A., Kuzhir P. (2019). Exploring thermal annealing and graphene–carbon nanotube additives to enhance crystallinity, thermal, electrical and tensile properties of aged poly(lactic) acid-based filament for 3D printing. Compos. Sci. Technol..

[20] Pastorek M., Kovalcik A. (2018). Effects of thermal annealing as polymer processing step on poly(lactic acid). Mater. Manuf. Process..

[21] Basgul C., Yu T., MacDonald D.W., Siskey R., Marcolongo M., Kurtz S.M. (2020). Does annealing improve the interlayer adhesion and structural integrity of FFF 3D printed PEEK lumbar spinal cages?. J. Mech. Behav. Biomed. Mater..

[22] Stojković J.R., Turudija R., Vitković N., Sanfilippo F., Păcurar A., Pleşa A., Ianoşi-Andreeva-Dimitrova A., Păcurar R. (2023). An experimental study on the impact of layer height and annealing parameters on the tensile strength and dimensional accuracy of FDM 3D printed parts. Materials.

[23] Simmons H., Tiwary P., Colwell J.E., Kontopoulou M. (2019). Improvements in the crystallinity and mechanical properties of PLA by nucleation and annealing. Polym. Degrad. Stab..

[24] Bhandari S., Lopez-Anido R.A., Gardner D.J. (2019). Enhancing the interlayer tensile strength of 3D printed short carbon fiber reinforced PETG and PLA composites via annealing. Addit. Manuf..

[25] Androsch R., Schick C., Di Lorenzo M.L. (2014). Melting of conformationally disordered crystals (α′-phase) of poly(L-lactic acid). Macromol. Chem. Phys..

[26] (2022). Standard Test Method for Tensile Properties of Plastics.

[27] Chacón J.M., Caminero M.A., García-Plaza E., Núñez P.J. (2017). Additive manufacturing of PLA structures using fused deposition modelling: Effect of process parameters on mechanical properties and their optimal selection. Mater. Des..

[28] Taguchi G., Chowdhury S., Wu Y. (2005). Taguchi’s Quality Engineering Handbook.

[29] (2022). Standard Test Method for Young’s Modulus, Tangent Modulus, and Chord Modulus.

[30] Murthy N.S., Minor H. (1990). General procedure for evaluating amorphous scattering and crystallinity from X-ray diffraction scans of semicrystalline polymers. Polymer.

[31] Wasanasuk K., Tashiro K., Hanesaka M., Ohhara T., Kurihara K., Kuroki R., Tamada T., Ozeki T., Kanamoto T. (2011). Crystal structure analysis of poly(L-lactic acid) α form on the basis of the 2-dimensional wide-angle synchrotron X-ray and neutron diffraction measurements. Macromolecules.

[32] Tănase M., Portoacă A.I., Diniță A., Brănoiu G., Zamfir F., Sirbu E.-E., Călin C. (2024). Optimizing mechanical properties of recycled 3D-printed PLA parts for sustainable packaging solutions using experimental analysis and machine learning. Polymers.

